# Meta-analysis of the prevalence of *Echinococcus* in dogs in China from 2010 to 2019

**DOI:** 10.1371/journal.pntd.0009268

**Published:** 2021-04-02

**Authors:** Qing-Long Gong, Gui-Yang Ge, Qi Wang, Tian Tian, Fei Liu, Nai-Chao Diao, Lan-Bi Nie, Ying Zong, Jian-Ming Li, Kun Shi, Xue Leng, Rui Du

**Affiliations:** 1 College of Chinese Medicine Materials, Jilin Agricultural University, Changchun, Jilin Province, People’s Republic of China; 2 College of Animal Science and Technology, Jilin Agricultural University, Changchun, Jilin Province, People’s Republic of China; 3 State Key Laboratory of Veterinary Etiological Biology, Key Laboratory of Veterinary Parasitology of Gansu Province, Lanzhou Veterinary Research Institute, Chinese Academy of Agricultural Sciences, Lanzhou, Gansu Province, People’s Republic of China; 4 Laboratory of Production and Product Application of Sika Deer of Jilin Province, Jilin Agricultural University, Changchun, Jilin Province, People’s Republic of China; PUCRS, BRAZIL

## Abstract

**Background:**

Echinococcosis (canine *Echinococcus* disease) is a neglected tropical disease that causes serious public harm. Dogs, as a terminal host of *Echinococcus* spp., are a key part of the *Echinococcus* epidemic. Echinococcosis spreads easily in humans and animals in some areas of China and it is therefore necessary to fully understand the prevalence of *Echinococcus* spp. in dogs.

**Methodology/Principal findings:**

PubMed, ScienceDirect, Chongqing VIP, China National Knowledge Infrastructure (CNKI), and WanFang databases were searched for relevant articles published in the past 10 years. A final total of 108 studies were included. The overall prevalence of *Echinococcus* spp. in dogs in China was 7.3%, with the highest point estimate found in sampling year 2015 (8.2%) and publication year 2015 (16.5%). Northwestern China (7.9%) had the highest infection rate in China. Qinghai Province (13.5%) showed the highest prevalence among the 11 provinces we included. We also found that geographical and climatic factors are related to the incidence of canine echinococcosis. We further investigated the source of heterogeneity by analysis of subgroups (sampling district, detection method, dog type, season, parasite species, medication, and study quality level).

**Conclusions/Significance:**

Our research indicated that *Echinococcus* spp. were still prevalent in some areas in China. More localized prevention and control policies should be formulated, including improving drinking water hygiene and strengthening hygiene promotion. We recommend the rational use of anti*-Echinococcus* drugs. In addition, treatment of livestock offal and feces and improving the welfare of stray dogs may play an important role in reducing canine *Echinococcus* infections.

## Author summary

Echinococcosis is a neglected zoonotic disease caused by the larval form of *Echinococcus* spp. tapeworms. It is prevalent in parts of China although unevenly distributed. The dog is one of the terminal hosts of *Echinococcus* spp., which is key in controlling the epidemic of canine echinococcosis. We constructed the first meta-analysis to assess the epidemic of *Echinococcus* in dogs in China over the past 10 years, and analyzed potential risk factors. We analyzed 108 studies of *Echinococcus* in dogs in China and evaluated the infection status and potential risk factors. The influential factors included province, geographical and climatic factors, publication year, sampling district category, detection methods, dog classification, medication, and the quality level of included studies. These important findings explain the differences in prevalence of canine echinococcosis in parts of China, and help to prevent the disease and reduce the risk to the public.

## Introduction

Echinococcosis is a zoonotic parasitic disease caused by the larval stages of cestodes of the genus *Echinococcus*. Echinococcosis, is one of the 20 neglected tropical diseases recognized by the World Health Organization (WHO). The distribution of this parasite is so widespread that it was included as one of a group of zoonoses by the WHO in its 2008–2015 strategic plan for the control of neglected tropical diseases [1,2].

Based on recent molecular and phylogenetic evidence, nine valid species have been found to belong to the *Echinococcus* genus, including *E*. *granulosus* sensu stricto (*E*. *granulosus* s.s., genotypes G1–G3), *E*. *equinus* (G4), *E*. *ortleppi* (G5), *E*. *canadensis* (G6–G10), *E*. *felidis*, *E*. *multilocularis*, *E*. *oligarthrus*, *E*. *shiquicus*, and *E*. *vogeli* [3,4,5–9]. *Echinococcus* spp. has two hosts in its life cycle. The first is the “definitive host” (mainly canine carnivores such as dogs, wolves, and foxes), and the second is the “intermediate host” (mainly sheep, cattle, camels, pigs, deer, and humans) [10]. *Echinococcus* disease in humans presents predominantly as the cystic (CE) or alveolar (AE) types [11], caused by *E*. *granulosus* and *E*. *multilocular*, respectively. AE and CE will cause serious harm to the human body, so both types are of major concern [12,13,14].

Although the treatment of echinococcosis has improved, reducing its definitive-host infection is still key to controlling the disease. Dogs play an important role in the life cycle of *Echinococcus* spp. as the definitive hosts. The eggs are swallowed by dogs, where they develop into adult worms in the intestines. The worms can live for about 5 months in the gut where they can cause intestinal parasitosis but do not harm other organs [15,16]. As the definitive host, dogs spread millions of parasite eggs on defecation and contaminate the external environment. Other herbivorous animals or humans becomes “intermediate host” for the parasite when they eat plant matter contaminated with these eggs [17]. Furthermore, 91% of herdsmen feed their dogs with sheep offal, 65% of sheep have close contact with dogs, and 47% of domestic dogs may be in direct contact with stray dogs. This suggests that the host is one of the most important risk factors for the spread of *Echinococcus* spp. [18,19].

China has one of the highest incidence rates of CE in the world. According to the investigation result, echinococcosis has a wide distribution, with almost 60 million residents at risk [10]. With the rapid development of the Chinese economy and continuous improvement of living standards, the number of families who keep pet dogs is increasing. At present, China has the largest number of dogs in the world: in 2012 there were 130 million and this number is growing [20]. The close relationship between dogs and humans makes it more difficult to prevent and control diseases. At present, echinococcosis is still a serious public health problem in northern and western China due to the methods of livestock rearing.

In the present study, we report the prevalence of *Echinococcus* spp. and perform a meta-analysis to understand the epidemiology over the last 10 years in China. We also assess the potential risk factors including sampling area (regions and provinces in China), geographical factors (longitude, latitude, and altitude), classification of dogs, detection methods, sampling time, and seasons and climate (annual temperature, maximum and minimum temperature, rainfall, and humidity) to determine which relate to *Echinococcus* prevalence in dogs.

## Materials and methods

### Search strategy

The study was conducted according to the PRISMA guideline (Preferred Reporting Items for Systematic Reviews and Meta-Analyses, [21]). A literature search was conducted for studies whose sampling timeframe was from 2010 to 2019. We included all studies published in English and Chinese on the prevalence of *Echinococcus* spp. in dogs across China. The databases searched included PubMed, ScienceDirect, Chongqing VIP, China National Knowledge Infrastructure (CNKI) and WanFang databases.

In PubMed, we used the Boolean operator “AND” to connect the MeSH words “Echinococcosis”, “Dogs”, and “China”; “OR” was used to connect the Entry Terms of the MeSH words. The final search formula was:

(Echinococcosis"[MeSH] OR *Echinococcoses* OR *Echinococcus* Infection OR *Echinococcus* Infections OR Infection, *Echinococcus* OR Cystic Echinocccosis OR Cystic *Echinocccoses* OR *Echinocccoses*, Cystic OR Echinocccosis, Cystic OR Hydatidosis OR Hydatidoses OR Cysts, Hydatid OR Cyst, Hydatid OR Hydatid Cysts OR Hydatid Cyst OR Hydatid Disease OR Hydatid Diseases OR *Echinococcus granulosus* Infections OR Granulosus Infections, *Echinococcus* OR *Granulosus* Infections, *Echinococcus* OR infection, *Echinococcus granulosus* OR infections, *Echinococcus granulosus*)

AND ("Dogs"[MeSH] OR Dog OR *Canis familiaris*)

AND ("China"[MeSH] OR People’s Republic of China OR Mainland China OR Manchuria OR Sinkiang OR Inner Mongolia)

In ScienceDirect, we use the advanced search to improve the accuracy of the results. The keywords “dog”, “Hydatid”, “Echinococcosis”, “prevalence”, and “China” were used; and the title, abstract or author-specified keywords had to contain the word “China”.

In the VIP database and CNKI, the topics were defined as “dog” AND “Hydatid” OR “dog” AND “*Echinococcus*” in Chinese in the advanced search.

In the WanFang database, the topic was defined as “canine hydatidosis” or “dogs *Echinococcus*” (in Chinese), and not “progress”, not “test”, not “a case”, and not “treatment” (in Chinese) in the advanced search. The final search formula in Chinese in the WanFang database was:

“Topic: (canine hydatidosis) + Topic: (dogs *Echinococcus*) ^ Topic: (progress) ^ Topic: (test) ^ Topic: (a case) ^ Topic: (treatment).

In the three Chinese databases, all of the retrieval processes included fuzzy searches or synonym expansion and all searches were conducted in Chinese.

We also visually scanned all reference lists from relevant studies to locate additional studies that may not have been identified by searching the electronic databases. We tried to contact the authors of the studies we could not download in the databases for additional information. No attempt was made to identify unpublished reports. We used Endnote (version X 9.3.1) to catalogue the articles retrieved.

### Inclusion and exclusion criteria

A preliminary selection of articles was conducted based on duplication, title, and abstract. Then we applied the following inclusion criteria:

- The study purpose was to examine the prevalence of echinococcosis in dogs in China;
- The study was published in English or Chinese;
- The study design was cross-sectional;
- The study contained the total number of dogs tested and the positive infection rate;
- The sampling year was reported and was 2010 or later;
- One sample was taken from each dog (not mixed samples).

**Fig 1:**
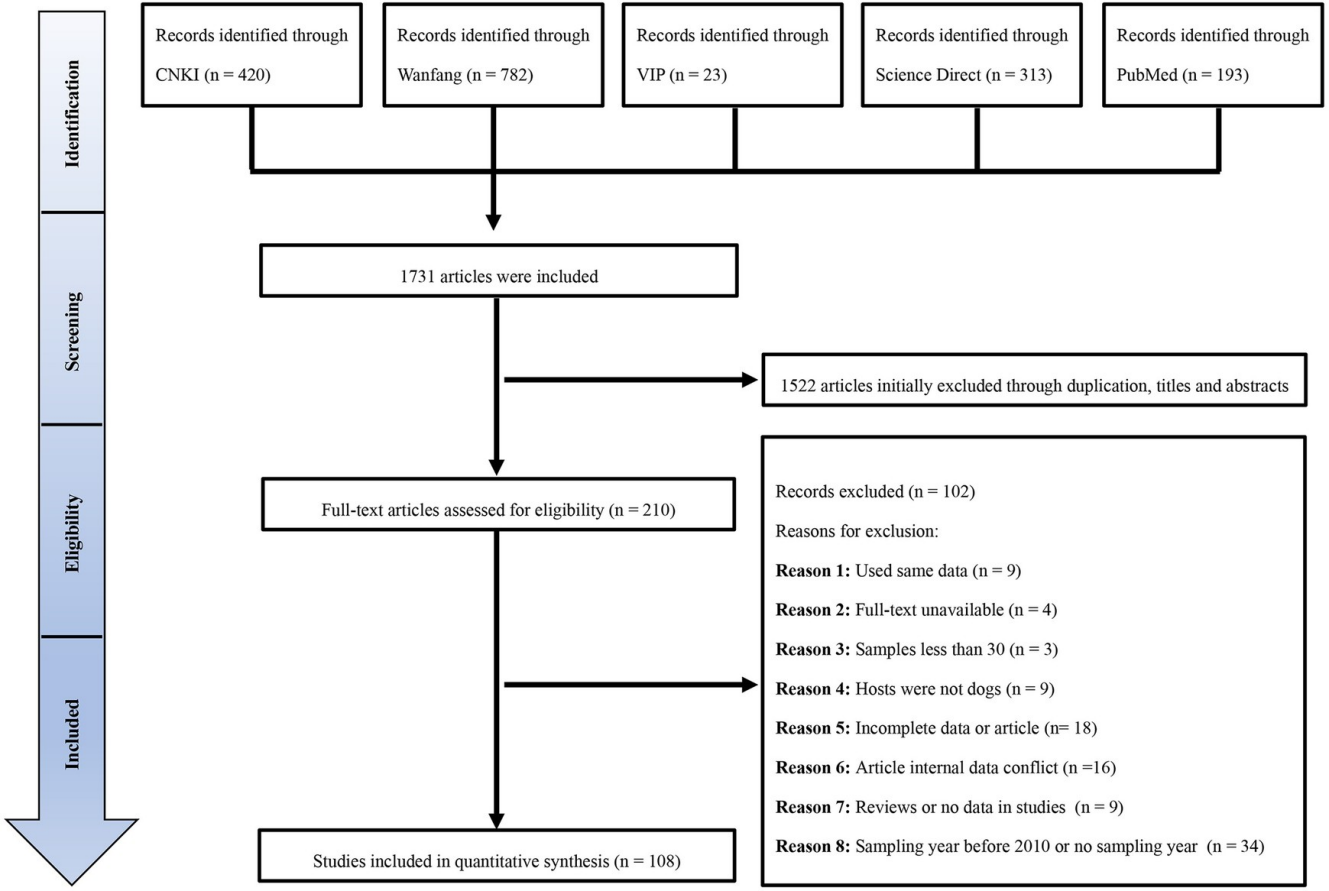
Flow diagram of searching for and selecting eligible studies.

The exclusion criteria: articles that were incompatible with the inclusion criteria were excluded from further consideration. The details of those excluded were presented in Fig 1.

### Data extraction

Five reviewers independently extracted and recorded data from each selected study. Any difference in opinion among reviewers or uncertainty about research eligibility was discussed with the author of this study and all reviewers had to extract data according to the result of the discussion. We extracted data from the included studies according to standardized data collection methods, using Microsoft Excel (version 16.32). The information recorded was as follows: first author, publication year, sampling year and season, geographical region of study, sampling district category (i.e. agricultural area, pastoral area, or urban area), detection methods (i.e. autopsy or ELISA), type of dog, medication, species of *Echinococcus*, the total number of dogs examined and the number of those with echinococcosis. We then obtained relevant geographic information. The meteorological data from the year involved was extracted from the China Meteorological Data Network. According to the sampling year, the following annual data were collected for each sampling location: altitude, rainfall, temperature, and maximum and minimum temperature. In addition, the longitude and latitude were noted.

### Quality assessment

We performed quality scoring based on the Grading of Recommendations Assessment, Development, and Evaluation methods (GRADE, [22]). Briefly, the following items were given 1 point when present:

- Random sampling;
- Detection method;
- Sampling number ≥ 200;
- Sample collection details;
- Four risk factors or more.

A total of 0 to 5 points were assigned to the articles: those with 0–1 points were defined as low quality; those with 2–3 points were considered moderate quality, and those with 4–5 points were deemed high quality.

### Statistical analysis

Our meta-analysis was executed by R software (R core team, version 4.0.0; R: A language and environment for statistical computing, 2018) where the “meta” package (version 4.12–0) was used to estimate models [23]. Before performing the meta-analysis, we chose the arcsine transformation (PAS) to convert the proportions to close to normal distribution. In a previous study, we discussed the selection criteria of the rate transformation in detail [24].

Cochran’s Q statistic and Higgin’s statistic were used to quantify the heterogeneity of studies. We selected the effects model according to the quantized heterogeneity in the included studies. Forest plots were used to express a pooled estimate of the included studies, the weight of each study and to display the heterogeneity between studies. In prevalence meta-analysis, strong heterogeneity can usually be predicted, so we prejudged the random-effects model for the overall estimation and subgroup analysis. We further used the symmetry of a funnel plot and an Egger’s test to judge whether there was publication bias or small sample size bias in our meta-analysis. A trim and fill analysis and a sensitivity analysis were used to assess whether our study was reliable [25].

We further tracked potential sources of heterogeneity by subgroup analysis and meta-regression analysis. We evaluated geographical region (Northwestern China vs. other regions), province (Qinghai Province vs. other provinces), sampling year (2010 vs. other sampling years), publication year (2015 vs. other years), sampling district category (urban vs. other district categories), detection method (autopsy vs. other methods), dog type (domestic dog vs. herding dog and stray dog), season (autumn vs. spring, summer, and winter), parasite species (*E*. *granulosus* vs. *E*. *multilocularis*), feeding model (intensive vs. extensive), medication (after drug vs. before drug), and study quality level (high vs. other quality levels).

We also assessed potential sources of heterogeneity by subgroup analysis of geographical factors. We evaluated latitude (25–30° vs. other latitudes), longitude (90–100° vs. other longitudes), altitude (6500 m vs. other altitudes), precipitation (≥ 1000 mm vs. other precipitation categories), humidity (≥ 70% vs. other humidity categories), mean temperature (≤ 5°C vs. mean temperature of other groups), lowest average temperature (≥ 5°C vs. lowest average temperature in other groups), and highest average temperature (≥ 20°C vs. highest average temperature in other groups). Our meta-analysis did not provide a review agreement and was not registered in Cochrane. The R software code we used in this meta-analysis is shown in S1 Table.

## Results

### Search results and eligible studies

We retrieved 1731 studies from the five databases. According to the inclusion criteria, 108 studies sampled between 2010 and 2019 were suitable for quantitative analysis (Fig 1). Only two papers were evaluated as low quality (0 or 1 points), 46 papers were classified as medium quality (2 or 3 points), and the remaining 60 papers were scored as high quality (4 or 5 points; S2 Table and S3 Table).

### Results of publication bias and sensitivity analyses

**Fig 2:**
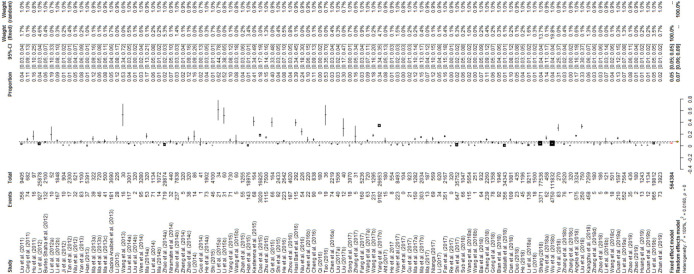
Forest plot of *Echinococcus* prevalence in dogs in China. The length of the horizontal line represents the 95% confidence interval, and the diamond represents the summarized effect.

We used PAS to convert the raw rate to ensure the data were closer to a normal distribution (Table 1). Consistent with our prediction, the results revealed a high heterogeneity in the included studies (*I*^*2*^ = 99.7%, *P =* 0.000; Fig 2).

**Table 1:** Normal distribution test for the normal rate and the different conversion of the normal rate.

Conversion form ^a^	*W*	*P*
**PRAW**	0.711	2.866e-13
**PLN**	NaN ^b^	NA ^c^
**PLOGIT**	NaN	NA
**PAS**	0.900	6.596e-7
**PFT**	0.878	5.854e-8

^a^ “PRAW”: original rate; “PLN”: logarithmic conversion; “PLOGIT”: logit transformation; “PAS”: arcsine transformation; “PFT”: double-arcsine transformation

^b^ “NaN”: meaningless number

^c^ “NA”: missing data.

**Fig 3:**
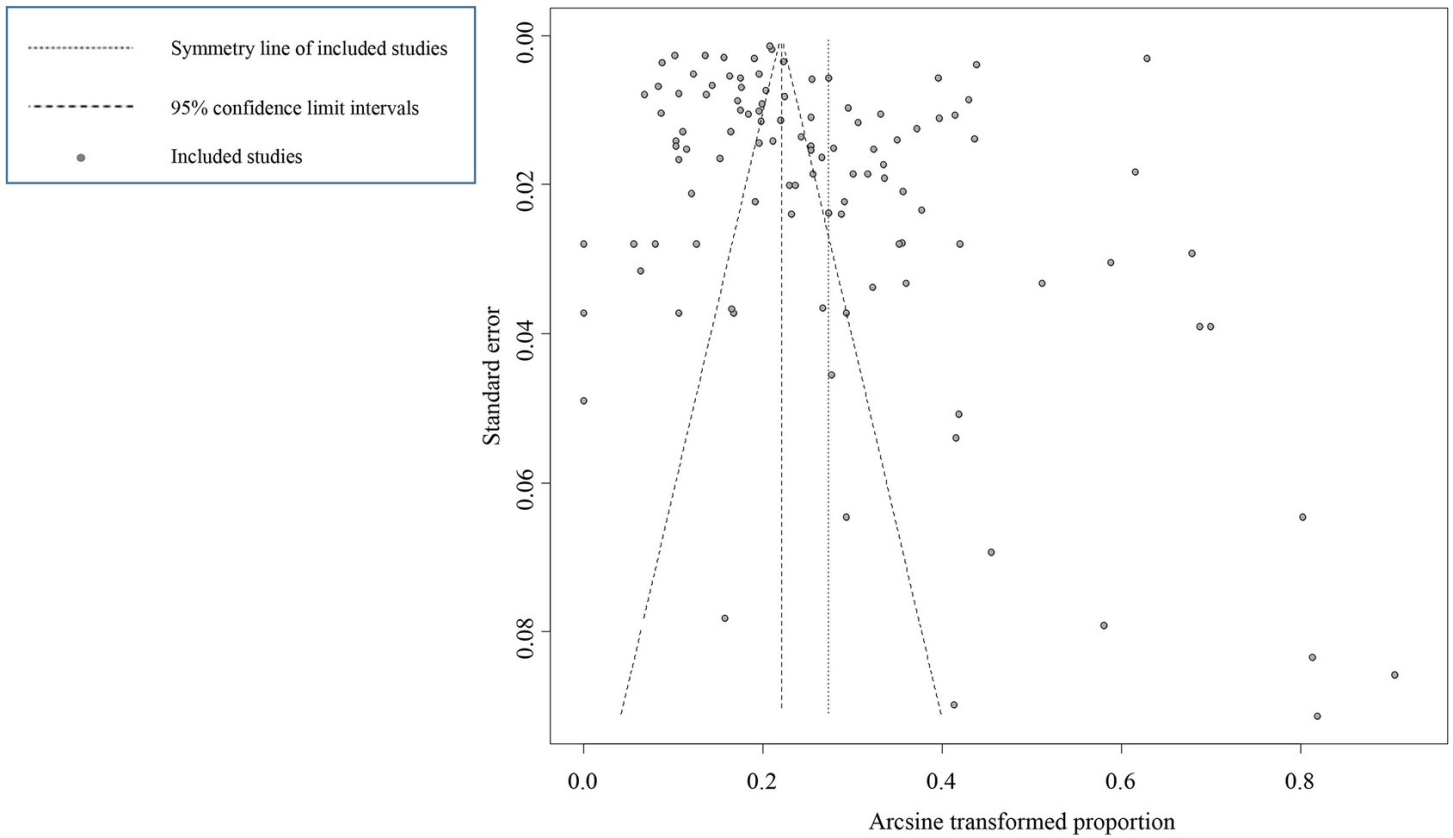
Funnel plot with pseudo 95% confidence interval for publication bias test.

The funnel chart was asymmetric, indicating that there might be small-sample size bias (or small-study effects bias) and/or publication bias in the included studies (Fig 3). The result of Egger’s test revealed that there was no publication bias in our meta-analysis (*P* > 0.05; S1 Fig, S4 Table). Therefore, there was small-study effects bias in our study. The result of the trim and fill test showed that there were 30 studies which were added (the point estimate was 0%) and the pooled estimate was finally changed (S2 Fig). Due to the small-sample size bias among the studies, the result of the trim and fill method may not be stable however, so this result should be treated with caution. We scored the quality of the included articles, and they lost points in the categories “no random sampling” (46 studies) and “sampling method details” (79 studies). This may bring heterogeneity to our research. The sensitivity analysis indicated that our results were reliable because the results were consistent with previous results when any of the studies were omitted (S3 Fig). Therefore, we believed that our meta-analysis was stable and reliable.

### Meta-analysis of *Echinococcus* spp. infection in dogs in China

The pooled prevalence of *Echinococcus* spp. infection in dogs in China was 7.3% (95% CI 6.1–8.6; 33,622/564,384) based on the data obtained from the selected suitable articles (Table 2). The pooled point estimate of *Echinococcus* infection in 2015 was 8.2% (95% CI: 2.9–15.8; 10,279/68,332). Although this was higher than other time periods, the difference was not significant (*P* > 0.05).

**Table 2:** Pooled prevalence of *Echinococcus* of dogs from 2010 to 2018 in China.

		No. studies	No. tested	No. positive	% (95% CI*)	Heterogeneity			Univariate meta-regression		
						χ^2^	P-value	I^2^ (%)	P-value	Coefficient (95% CI)	R^2^*
**Sampling years**									0.185	0.116 (-0.056 to 0.288)	0.55%
	2010	15	34,765	2,117	8.0% (6.0–10.2)	562.11	< 0.01	97.5%			
	2011	20	67,811	5,765	7.2% (4.6–10.3)	3409.64	< 0.01	99.4%			
	2012	34	72,596	3,517	6.6% (4.8–8.6)	3275.96	< 0.01	99.0%			
	2013	19	46,532	1,669	6.3% (4.2–8.9)	1672.76	< 0.01	98.9%			
	2014	10	28,214	787	6.7% (4.1–10.1)	626.66	< 0.01	98.6%			
	2015	21	68,332	10,279	8.2% (2.9–15.8)	16444.03	< 0.01	99.9%			
	2016	23	63,453	2,390	5.7% (4.2–7.3)	1387.06	< 0.01	98.4%			
	2017	14	19,955	635	4.7% (2.3–7.8)	760.83	< 0.01	98.3%			
	2018	3	2,133	26	1.7% (0.5–3.6)	11.39	< 0.01	82.4%			
**Total**		108	564,384	33,622	7.3% (6.1–8.6)	33444.14	0.000	99.7%			

CI*: Confidence interval

R^2^: Proportion of inter-study variance explained.

Infection rates varied across different geographical regions in China. In the region subgroups, the highest point estimate was in northwestern China (7.2%, 95% CI: 6.2–8.2; 16,562/391,161). At the province level, the lowest prevalence was in Shaanxi Province (0.0%, 95% CI: 0.0–0.2; 0/640; Table 3).

**Table 3:** Pooled prevalence of *Echinococcus* in dogs in different areas in China.

		No. studies	No. tested	No. positive	% (95% CI)	Heterogeneity			Univariate meta-regression		
						χ^2^	P-value	I^2^ (%)	P-value	Coefficient (95% CI)	R^2^
**Region** ^**a**^									0.102	-0.045 (-0.098 to 0.009)	26.38%
	Eastern China	1	1,243	13	1.1% (0.6–1.7)	0.00	-	-			
	Northern China	8	60,085	1,430	4.8% (3.4–6.5)	291.82	< 0.01	97.6%			
	Northwestern China	87	391,161	16,562	7.2% (6.2–8.2)	11452.46	0.00	99.2%			
	Southwestern China	20	111,895	15,617	5.5% (2.3–10.0)	14287.04	0.00	99.9%			
**Province**									0.000	-0.150 (-0.208 to -0.091)	50.09%
	Gansu	17	142,013	6,572	4.1% (2.9–5.5)	1695.71	< 0.01	99.1%			
	Hebei	1	121	9	7.4% (3.5–12.8)	0.00	-	-			
	Inner Mongolia	7	59,964	1,421	4.6% (3.2–6.3)	284.24	< 0.01	97.9%			
	Jiangsu	1	1243	13	1.1% (0.6–1.7)	0.00	-	-			
	Ningxia	7	24,431	1,461	6.7% (2.7–12.2)	1243.83	< 0.01	99.5%			
	Qinghai	26	24,552	2,972	13.5% (10.8–16.4)	713.03	< 0.01	96.5%			
	Shaanxi	1	640	0	0.0% (0.0–0.2)	0.00	-	-			
	Sichuan	5	56,428	12,596	9.4% (1.3–23.6)	7308.71	< 0.01	99.9%			
	Tibet	9	28,397	1,934	6.9% (5.8–8.1)	92.78	< 0.01	91.4%			
	Xinjiang	37	200,165	5,557	5.8% (4.7–7.0)	3960.49	< 0.01	99.1%			
	Yunnan	5	26,430	1,087	2.2% (0.6–4.6)	231.42	< 0.01	98.3%			

^a^ Region: Northern China: Hebei, Inner Mongolia; Northwestern China: Shaanxi, Gansu, Qinghai, Ningxia, Xinjiang; Eastern China: Jiangsu; Southwestern China: Yunnan, Sichuan, Tibet.

We also analyzed geographical factors and found that the highest prevalence was in latitude 30–35° (8.7%, 95% CI: 6.9–10.7; 3,519/30,669), longitude 90–100° (6.2%, 95% CI: 5.0–7.4, 3,745/46,021), and at altitudes ≥ 6500 m (21.6%, 95% CI: 12.0–32.9; 580/4,514). According to the analysis of the temperature in each sampling year, we found that the highest prevalence was at mean temperatures ≤ 5°C (7.5%, 95% CI: 6.0–9.2, 4,443/60,892; Table 4). In the sampling district subgroup, the prevalence in pastoral areas (14.8%, 95% CI: 10.1–20.3; 924/13,697) was significantly higher (*P* < 0.05) than in other areas (Table 5).

**Table 4:** Subgroup analysis of geographical and climatic factors.

		No. studies	No. tested	No. positive	% (95% CI)	Heterogeneity			Univariate meta-regression	
						χ^2^	*P* value	*I^2^* (%)	*P* value	Coefficient (95% CI)
**Latitude**									0.013	-0.041 (-0.073 to -0.09)
	25–30°	11	34,304	1,844	4.1% (3.4–4.8)	720.58	< 0.010	88.9%		
	30–35°	17	30,669	3,519	8.7% (6.9–10.7) **	1378.88	< 0.010	97.0%		
	35–40°	44	69,854	4,583	5.5% (4.5–6.6)	4718.67	< 0.010	97.0%		
	40–45°	23	67,240	2,520	5.3% (4.1–6.6)	2226.45	< 0.010	97.8%		
	≥ 45°	20	82,492	3,474	5.0% (3.2–7.2)	5253.08	< 0.010	99.4%		
**Longitude**									0.184	0.021 (-0.010 to 0.052)
	100–110°	44	79,423	5,436	5.4% (4.5–6.4)	5418.50	< 0.010	97.2%		
	≥ 110°	9	12,333	481	4.3% (2.5–6.5)	650.04	< 0.010	96.5%		
	70–90°	36	146,782	6,278	5.2% (4.1–6.4)	7696.52	< 0.010	98.9%		
	90–100°	22	46,021	3,745	6.2% (5.0–7.4) **	3273.69	< 0.010	97.4%		
**Altitude**									0.000	0.229 (0.144 to 0.314)
	1500–2500 m	31	47,185	4,018	6.2% (5.0–7.6)	2978.69	< 0.010	96.7%		
	2500–3500 m	14	23,653	853	3.7% (2.5–5.1)	841.06	< 0.010	95.5%		
	3500–4500 m	12	27,859	2,948	7.4% (6.1–8.7)	908.70	< 0.010	93.4%		
	4500–6500 m	4	5,593	473	6.9% (4.7–9.5)	99.73	< 0.010	92.0%		
	≥ 6500 m	8	4,514	580	21.6% (12.0–32.9) **	458.17	< 0.010	98.0%		
	≤ 700 m	18	128,502	3,045	2.7% (1.9–3.6)	1983.74	< 0.010	98.7%		
	700–1500 m	43	112,879	5,389	4.4% (3.5–5.4)	5848.00	< 0.010	98.2%		
**Precipitation**									0.003	-0.093 (-0.155 to -0.032)
	≥ 1000 mm	7	7,897	270	2.3% (1.4–3.5)	112.63	< 0.010	85.8%		
	≤ 200 mm	39	141,092	5,393	4.7% (3.7–5.8)	5358.51	< 0.010	98.8%		
	200–500 mm	59	103,412	6,858	6.6% (5.5–7.7) **	7549.60	< 0.010	97.8%		
	500–1000 mm	25	56,264	3,769	5.0% (3.8–6.2)	3252.33	< 0.010	97.5%		
**Humidity**									0.001	-0.151 (-0.237 to -0.065)
	30–50%	7	96,184	3,890	4.2% (3.4–5.2)	3792.07	< 0.010	97.4%		
	50–60%	71	201,170	1,0458	6.1% (5.2–7.1)	10768.04	< 0.010	98.6%		
	60–70%	22	46,115	2,565	6.2% (4.8–7.8) **	2942.77	< 0.010	97.5%		
	≥ 70%	3	2,787	37	0.9% (0.3–1.9)	25.66	< 0.010	72.7%		
**Mean temperatur**									0.001	0.055 (0.023 to 0.086)
	10–15°C	16	25,870	1,240	3.7% (2.6–5.0)	1316.14	< 0.010	96.0%		
	≥ 15°C	4	10,735	445	2.3% (1.3–3.8)	287.27	< 0.010	93.7%		
	≤ 5°C	34	60,892	4,443	7.5% (6.0–9.2) **	3953.86	< 0.010	98.1%		
	5–10°C	73	215,967	1,0420	5.5% (4.7–6.3)	11241.44	< 0.010	98.3%		
**Lowest average temperature**									0.002	-0.053 (-0.087 to -0.020)
	≤ -5°C	13	17,698	2,036	9.2% (7.4–11.3) **	388.37	< 0.010	94.3%		
	-5–0°C	36	54,187	3,172	6.8% (5.5–8.2)	3467.02	< 0.010	97.4%		
	0–5°C	65	200,187	9,606	5.1% (4.3–6.0)	9985.84	< 0.010	98.5%		
	≥ 5°C	21	37,296	1,612	3.7% (2.7–4.9)	2061.40	< 0.010	96.7%		
**Highest average temperature**									0.003	-0.078 (-0.129 to -0.028)
	≤ 10°C	15	30,916	2,968	8.4% (6.3–10.0) **	1408.62	< 0.010	96.9%		
	10–15°C	56	167,661	7,819	7.1% (5.9–8.3)	9484.28	< 0.010	98.7%		
	15–20°C	51	97,727	5,040	4.1% (3.4–4.9)	4808.12	< 0.010	97.1%		
	≥ 20°C	7	12,875	587	2.7% (1.7–4.1)	397.02	< 0.010	94.0%		

**Table 5:** Other potential risk factors of dogs *Echinococcus* infection in China.

		No. studies	No. tested	No. positive	% (95% CI*)	Heterogeneity			Univariate meta-regression		
						χ^2^	P-value	I^2^ (%)	P-value	Coefficient (95% CI)	R^2^
**Sampling district category**									0.018	0.105 (0.019 to 0.192)	0.00%
	Semi-agricultural pastoral area	8	14,764	540	4.5% (2.6–7.0)	182.12	< 0.01	96.2%			
	Agricultural area	6	15,774	409	3.4% (1.8–5.3)	75.57	< 0.01	93.4%			
	Pastoral area	16	13,697	924	14.8% (10.1–20.3)	869.76	< 0.01	98.3%			
	Urban	5	9,315	234	3.5% (1.8–5.9)	72.15	< 0.01	94.5%			
Detection methods*									< 0.001	0.347 (0.266 to 0.427)	-
	ICGT	1	52	10	19.2% (9.8–30.9)	0.00	-	-			
	Autopsy	7	164	57	26.3% (11.7–44.2)	33.45	< 0.01	82.11%			
	ELISA	98	557,353	32,254	5.9% (4.8–7.1)	31692.36	0.00	99.7%			
	Flotation method (NaCl)	5	197	83	40.3% (26.1–55.5)	18.55	< 0.01	78.4%			
	Multiplex-PCR	2	4,074	825	24.8% (11.0–41.9)	85.17	< 0.01	98.8%			
**Dogs classification**									< 0.001	0.103 (0.061 to 0.144)	14.90%
	Domestic dog	73	463,699	16,872	4.9% (4.2–5.6)	7293.33	0.00	99.0%			
	Herding dog	6	9,743	651	18.5% (6.9–34.0)	157.78	< 0.01	96.8%			
	Stray dog	10	2,705	334	7.4% (5.0–10.2)	153.71	< 0.01	94.1%			
Season*									0.795	0.017 (-0.113 to 0.147)	15.57%
	Spring	8	2,181	228	11.2% (4.6–20.2)	223.04	< 0.01	96.9%			
	Summer	9	7,790	782	10.2% (6.8–17.2)	355.98	< 0.01	97.8%			
	Autumn	6	3,708	148	10.0% (4.6–17.2)	77.11	< 0.01	93.5%			
	Winter	3	396	28	10.9% (0.0–39.4)	82.53	< 0.01	97.6%			
Parasite species*									0.638	0.031 (-0.100 to 0.163)	30.96%
	*E*. *granulosus*	43	104,093	3,740	7.3% (5.9–8.9)	3013.88	0.00	98.6%			
	*E*. *multilocularis*	2	3,914	266	9.0% (2.2–19.8)	61.00	< 0.01	98.4%			
**Medication**									0.022	0.266 (0.039 to 0.493)	3.63%
	Before drug	3	278	61	22.2% (9.4–38.5)	11.48	0.00	82.6%			
	After drug	10	34,254	1,601	4.8% (1.4–10.2)	2103.52	< 0.01	99.6%			
**Quality level**									0.214	-0.028 (-0.072 to 0.016)	22.01%
	Low	2	2,354	364	11.5% (4.1–21.8)	14.15	-	92.9%			
	Middle	46	117,648	15,910	8.2% (5.1–11.8)	17318.96	0.00	99.7%			
	High	60	444,382	17,348	6.2% (5.4–7.2)	7709.30	0.00	99.2%			

Method*: ICGT: Immune colloidal gold technique; Flotation: Flotation method (NaCl).

Season*: Spring: Mar. to May.; Summer: Jun. to Aug.; Autumn: Sep. to Nov.; Winter: Dec. to Feb.

A total of four detection methods were used in the included studies. The flotation method (NaCl) showed the highest detection rate (40.3%, 95% CI: 26.1–55.5; 83/197) of the four methods. The different types of dogs showed a significantly different (P < 0.05) infection rate: herding dogs were most often infected, with a prevalence of up to 18.5% (95% CI: 6.9–34.0; 651/9,743). The prevalence of *E*. *multilocularis* infection (9.0%, 95% CI: 2.2–19.8; 266/3,914) was slightly higher than for *E*. *granulosus* (7.3%, 95% CI: 5.9–8.9; 3,740/104,093), but the difference was not significant (P > 0.05). We also conducted other subgroup analyses such as sampling season (summer; 10.2%, 95% CI: 6.8–17.2), medication (before administration; 22.2%, 95% CI: 9.4–38.5), and quality level (high; 6.2%, 95%CI: 5.4–7.2) of the included studies, and the results are shown in Table 5.

The univariate meta-regression showed that province, publication year, sampling district, detection method, dog classification, medication, latitude, altitude, precipitation, humidity, mean temperature, lowest average temperature, and highest average temperature may be major sources of heterogeneity (*P* < 0.05).

## Discussion

Echinococcosis is a serious neglected zoonotic disease with a worldwide prevalence. Cystic echinococcosis is found in all continents except Antarctica [26]. Infection is a serious problem in parts of China, especially in rural populations of western China. This disease has a significant impact on people’s health and it is therefore under close scrutiny by the Chinese Ministry of Health [27,28]. Due to lack of understanding of transmission dynamics and a lack of effective control strategies for the disease, morbidity and mortality have increased [29]. Dogs play an important role in the spread of the disease as definitive hosts. In China, the number of pet dogs at the end of 2018 was 74 million and there are a large number of stray dogs. Therefore, the high positive rate of *Echinococcus* may be caused by the large number of dogs and their close contact with humans. Although there have been numerous investigations of *Echinococcus* in dogs, the present study is the first meta-analysis of *Echinococcus* prevalence in dogs in China. We found that the overall prevalence rate was 7.3%, which is lower than in Iran (pooled prevalence 14.61%, [30]), but higher than for the European Union and adjacent countries (pooled prevalence 0.3%, [31]).

The prevalence of *Echinococcus* was highest in dogs in northwestern China. No statistical differences were found in the regional subgroups (*P* > 0.05), although further regression analysis of the province subgroups did show significant differences (*P* < 0.05). We found a high heterogeneity between region and province subgroups, so we used provinces and regions as covariates and conducted a joint analysis of these two subgroups. This analysis explained 26.38% of the heterogeneity (t^2^ = 0.012, R^2^ = 26.38%). We found that the infection rate of *Echinococcus* in Qinghai, Tibet and Sichuan was significantly higher than in other provinces. Latitude, longitude and altitude analysis also showed the same results (Qinghai-Tibet Plateau and Sichuan are located at east longitude 73°19′ to 108°12′ and north latitude 26°00′ to 39°47′, and the altitude is > 3500 m).

The factors that lead to regional differences are complex and diverse. In Northwestern China, Qinghai Province is a mainly pastoral area, and the living environment is relatively complex compared with other areas. Although the Chinese government has begun to solve the water safety problems of herders [32], herding dogs or livestock may still drink water in a nearby river or excrete near the river, resulting in *Echinococcus* infection [33]. Secondly, they have more opportunities for intimate contact with other domestic animals, and some herders feed their dogs with uncooked giblets from sheep and other domestic animals [34]. Sheep have an especially high infection rate, which increases the chance of dogs becoming infected with *Echinococcus*. Dog feces are generally discharged into the pasture without treatment, which may cause the environment to be contaminated by *Echinococcus*. Therefore, promotion of echinococcosis prevention and control should be carried out to increase awareness for herders, as well as prohibiting the sale of sick animal offal and feeding it to dogs. These methods may help disease prevention and control.

In the Sichuan-Tibet region, many stray dogs have been adopted by families or temples due to nomadic life and religious beliefs. In addition, Buddhism advocates the natural death of old livestock, increasing the chance of old animals suffering from echinococcosis [35]. Unrestricted handling of livestock offal has also increased the risk of *Echinococcus* eggs being ingested by dogs, resulting in a higher infection rate than in other regions. According to previous research, there is a negative correlation between altitude and economy [36]. In addition, we found that echinococcosis was positively and significantly correlated with altitude [37]. This implies that *Echinococcus* infection may be negatively correlated with economic development [38]. Therefore, formulating more targeted plans according to cultural and economic differences at high altitude may play a positive role in preventing canine and human echinococcosis.

It is worth noting that the studies included only investigated prevalence in certain regions (Eastern China, Northern China, Northwestern China, and Southwestern China). This strongly indicated that the *Echinococcus* infection was regional in China [10,39]. According to the statistics of the National Health Commission from 2018 to 2019, there were 4003 cases of echinococcosis and 2 deaths in China [40]. It can be suspected that some of these cases were caused by *Echinococcus* in dogs. The public health threats of canine echinococcosis should not be ignored. Reducing the rate of canine echinococcosis is very important in reducing the incidence of human echinococcosis. It is recommended to maintain continuous and intensive epidemiological monitoring to clarify the true infection rate of canine echinococcosis in various regions of China.

The classification of dog types and sampling locations confirmed that the infection rate in pastoral areas is almost three times that in other areas. The infection rate in herding dogs is also significantly higher than in stray and domestic dogs. This is the same as our speculation in the regional subgroup. In addition, the reason why *Echinococcus* prevalence in herding dogs was higher than other types may be that these dogs are known to feed on voles, pikas and hares. Studies have found that several small mammal species, such as the Qinghai vole (*Microtus fuscus*) and the Plateau pika (*Ochotona curzoniae*), are known to act as intermediate hosts for *E*. *multilocularis*. Among them, the plateau pika is considered particularly important [41,42]. It is difficult to carry out large-scale periodic deworming activities on stray dogs due to lack of control, so the infection rate in stray dogs is substantially higher than in domestic dogs. Stray dogs, especially males, often patrol their territory, meaning that their range of activity is wider than domestic dogs and therefore they are at a greater risk of *Echinococcus* infection.

Interestingly, we found that infection rates in agricultural areas and urban areas were similar. This may be due to the large number of stray dogs and cats in the city, and these dogs have a larger range of activity and more opportunities for exposure to parasites. In some agricultural areas of China, dogs are usually used as guard dogs or as household pets and are prevented from roaming freely; this means they have less chance of finding and ingesting raw carcass meat and offal [19]. Therefore, we should strictly control stray dogs, perform a strict registration of domestic dogs, carry out regular deworming and limit the scope of activities. Since we did not have enough data on the sex and age of dogs, we did not analyze this aspect. Therefore, the relationship between gender and *Echinococcus* infection needs further study.

In our study, the prevalence of *Echinococcus* infection in dogs in China gradually declined from 2010 to 2018. This may be due to the further implementation of the "National Mid- and Long-Term Animal Disease Control Plan (2012–2020)" and the "National Echinococcosis and Other Key Parasitic Disease Control Plans (2016–2020)". To the best of our knowledge, echinococcosis is still an ongoing epidemic in some areas of China. As the Chinese government has stepped up efforts to prevent and control the epidemic in these areas, various policies have been gradually implemented. As a result, the infection rate in high-risk areas such as Qinghai, Sichuan, and Tibet have shown a downward trend. Therefore, targeted prevention and control policies and comprehensive implementation may be the key to prevention and control of echinococcosis. It was worth noting that the prevalence of *Echinococcus* in dogs has increased since 2015. We found that the 95% CI of the 2015 group was larger than that of the other groups. We further reviewed the studies in this group and found that the prevalence was higher than 20% in all of these 6 studies. There were 3 studies with a sample size of less than 100 (86, 40, and 36, respectively; Wu et al. (2018a), Liu (2017b), and Tao (2016) in S2 Table). Therefore, the high prevalence in 2015 may have been caused by small sample bias.

In a prevalence meta-analysis, different detection methods are usually the main source of heterogeneity. Therefore, we used multiple meta-regression to trace the potential association between detection methods and other potential risk factors. The results showed that up to 50% of the heterogeneity could be explained by detection method. In our study, the studies used different methods for identifying the infection rate of *Echinococcus*. Detection rate was lowest in the most commonly-used method (ELISA). Although this method is easy to use, has a good sensitivity, and is suitable for large-scale testing, there is a large human factor. In addition, test results vary between manufacturers, and cross-reaction with other *Taenia* species or other helminth antigens can lead to false positives [43]. Testing by autopsy is the most reliable method [35]. Although this method is highly sensitive, it is laborious, raises ethical issues, and is biohazardous [44]. In addition, it is impossible to sacrifice dogs for detection purposes in some areas due to religious and cultural reasons. The positive rate of the flotation (NaCl) method was 40.3%, which may be because eggs of all species of the family Taenidae (genera *Echinococcus* and *Taenia*) are morphologically indistinguishable from one another [45]. In addition, although the multiplex-PCR method has higher reliability, it is only suitable for laboratory testing due to the complicated operation and high cost. The arecoline purgation method was often used in the past. However, due to the high risk, the degree of harm to dogs and the low sensitivity, it has seen less use in recent years [43,46]. Therefore, the joint use of two methods may improve the detection rate of *Echinococcus*. At the same time, it is also important to develop more accurate, simple and low-cost methods.

There was no significant difference between season subgroups. However, we found that the prevalence of *Echinococcus* infection is negatively correlated with the ambient temperature and positively correlated with the ambient humidity within a certain range. From the three temperature subgroups, we found that eggs of *Echinococcus* seem more likely to survive at low temperatures. Veit et al. (1995) [47] found that eggs of *E*. *multilocularis* could survive in the wild for up to 240 days with no loss of viability, even when exposed to temperatures of -10°C, -20°C, and -30°C. However, a temperature of 43°C rapidly kills off the eggs irrespective of the degree of humidity. This shows that *Echinococcus* eggs are more likely to remain infectious under relatively humid and low temperature conditions [47]. Therefore, strengthening the prevention and control of canine echinococcosis during the cold and wet seasons (typically spring and winter in China) may help reduce the spread of *Echinococcus* in dogs.

We conducted a subgroup analysis of the *Echinococcus* species, but the difference was not significant. It is worth noting that only two studies detected *E*. *multilocularis*. This also implies that the main *Echinococcus* species in China may be *E*. *granulosus* [48].

We also analyzed the rate of whether animals had been treated. As expected, the positive rate was higher before drug administration than after. The most commonly-used drug in China is praziquantel, and there are encouraging results of using praziquantel as insect repellent [49]. Praziquantel drugs have achieved greater effects abroad [50]. We also need to further strengthen the research on vaccines and drugs, hoping to obtain a better therapeutic method.

The advantage of our research lies in the large sample size, the wide area, the long-term investigation period, the relatively complete range of potential risk factors and the correct methodology. However, our meta-analysis still had several limitations. First, we tried to identify all studies related to *Echinococcus* infection in the selected databases by searching with several different MeSH terms. However, these searches may not have found all the relevant studies. Second, the lack of research in some subgroups or small sample sizes may lead to inaccurate estimates of this subgroup. Third, most of the included studies did not state whether random sampling was used, which may lead to heterogeneity. Fourth, few studies were conducted in 2018, and there were no studies from 2019. However, the data shows that in 2019 there were 4003 new echinococcosis patients in China and some of these infections may originate from dogs. This shows that *Echinococcus* in dogs still exists in China. It is recommended to strengthen the monitoring of canine *Echinococcus* to reduce the risk of human infection. Fifth, the potential risk factors we extracted may not be complete. The included articles do not mention the age and sex of the dog, which may be important infection factors. Sixth, this study was not registered; however, it was carried out strictly in accordance with PRISMA guidelines.

The high prevalence of *Echinococcus* in dogs in mainland China may present a high risk of human infection. To improve public health, proper management and monitoring measures of *Echinococcus* infection in dogs are necessary. Disease control policies may play an active role in prevention. *Echinococcus* infection is affected by dog type and area. In addition, echinococcosis is negatively correlated with temperature, and positively correlated with altitude and with humidity within a certain range. Therefore, adopting relevant measures in different regions may help to reduce the local infection rate. The research provides epidemiological data and a theoretical basis for further formulating *Echinococcus* in dogs prevention and a control plan.

## Supporting information

S1 Fig Egger’s test for publication bias. (TIF)

S2 Fig Funnel plot with trim and fill analysis for the publication bias test. (TIF)

S3 Fig Sensitivity analysis. After removing one study at a time, the remaining studies were re-combined using a random-effects model to verify the impact of a single study on the overall results.
(TIF)

S4 Fig Multivariate meta-regression analysis for publication years. (TIF)

S1 Table The code in R for this meta-analysis. (DOCX)

S2 Table Included studies on *Echinococcus* infection in dogs in China. (DOCX)

S3 Table The quality scores and the literature list in this meta-analysis. (DOCX)

S4 Table Egger’s test for publication bias. (DOCX)

S5 Table PRISMA Checklist for meta-analysis. (DOCX)
