# Supplementary figures and images for: Meta-analysis of the prevalence of Echinococcus in dogs in China from 2010 to 2019
Source: PLoS Negl Trop Dis. 2021 Apr 2;15(4):e0009268. doi: 10.1371/journal.pntd.0009268 (PMC8018629; doi:10.1371/journal.pntd.0009268)

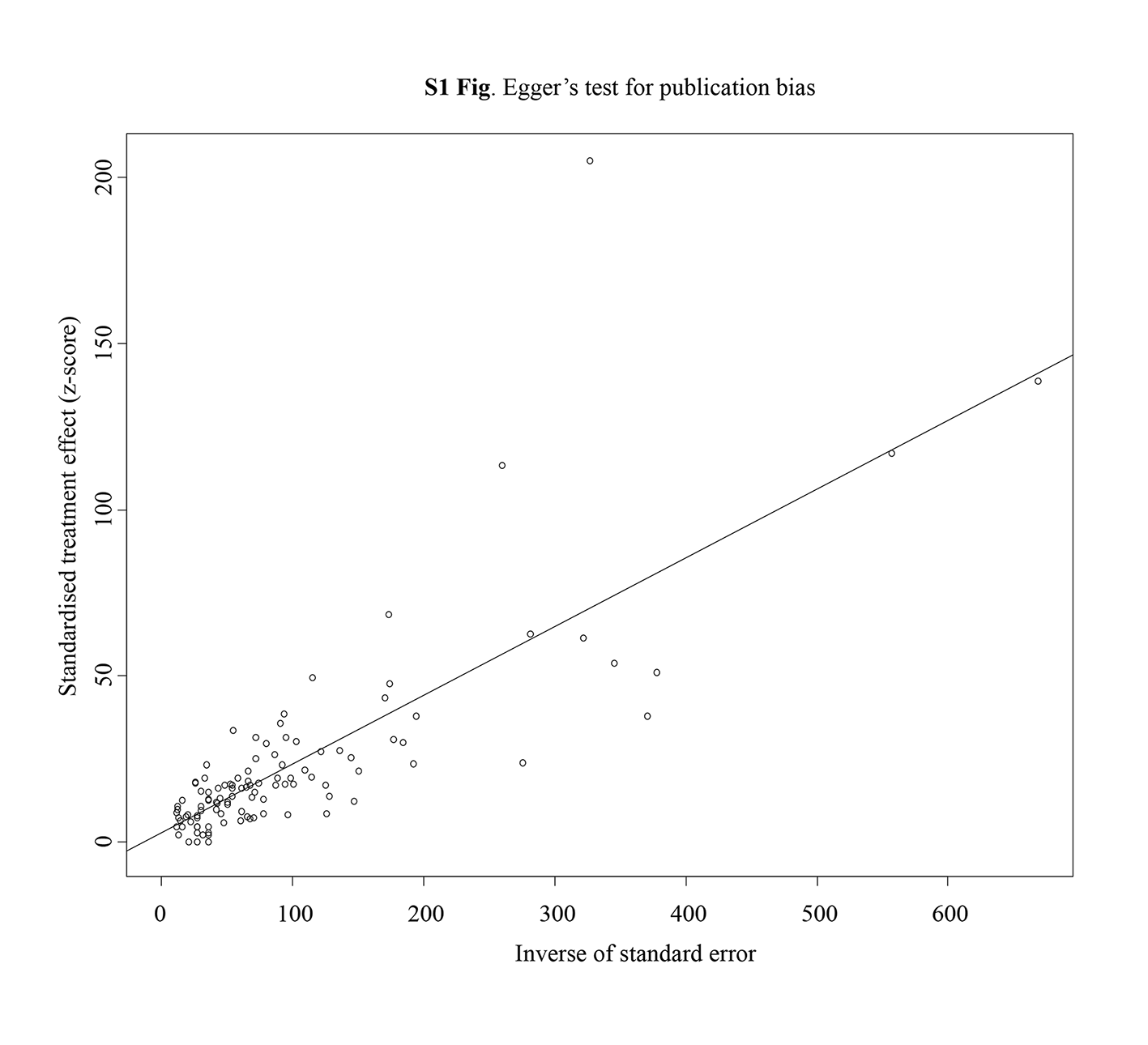

Supplement: S1 Fig — (TIF) [file pntd.0009268.s001.tif]

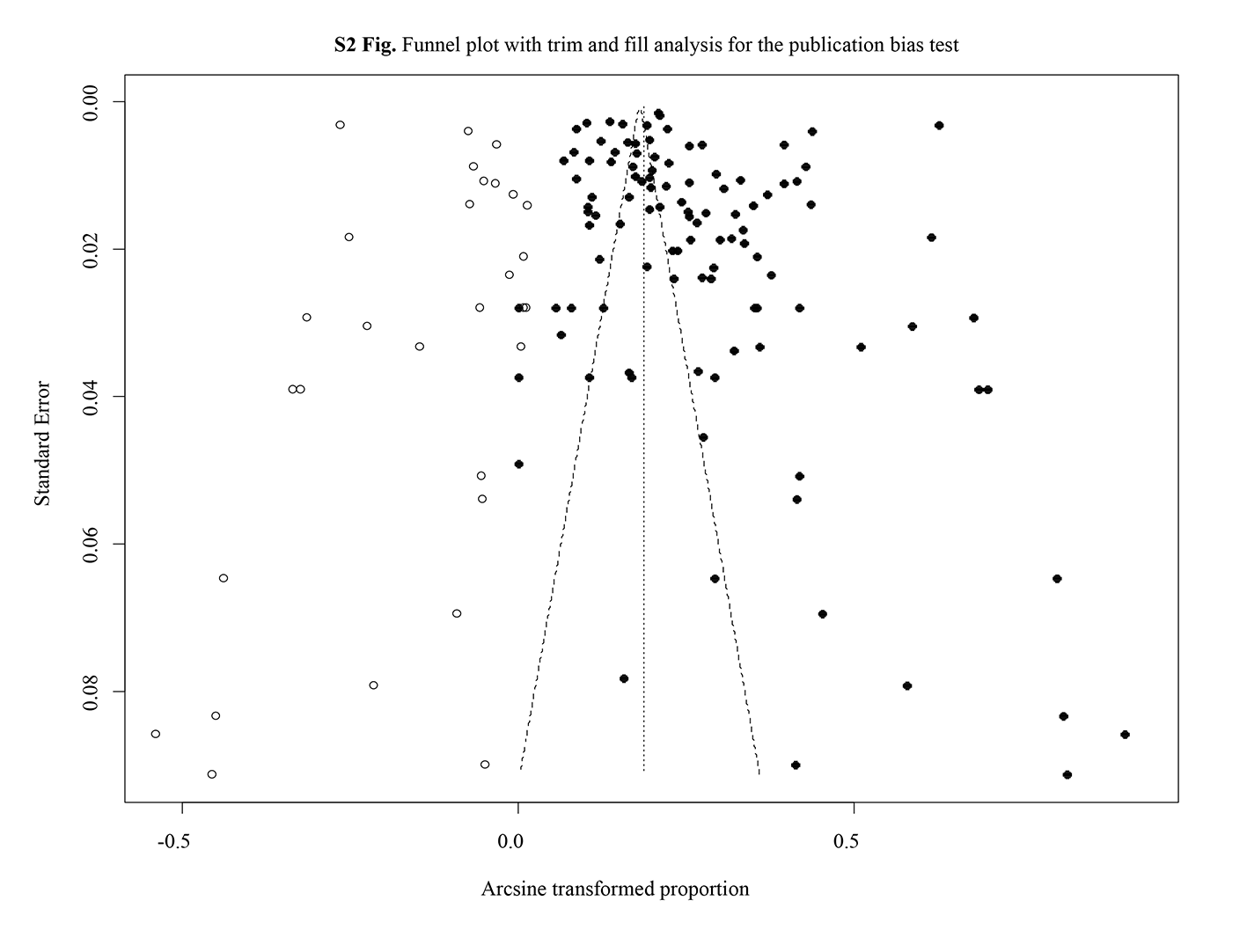

Supplement: S2 Fig — (TIF) [file pntd.0009268.s002.tif]

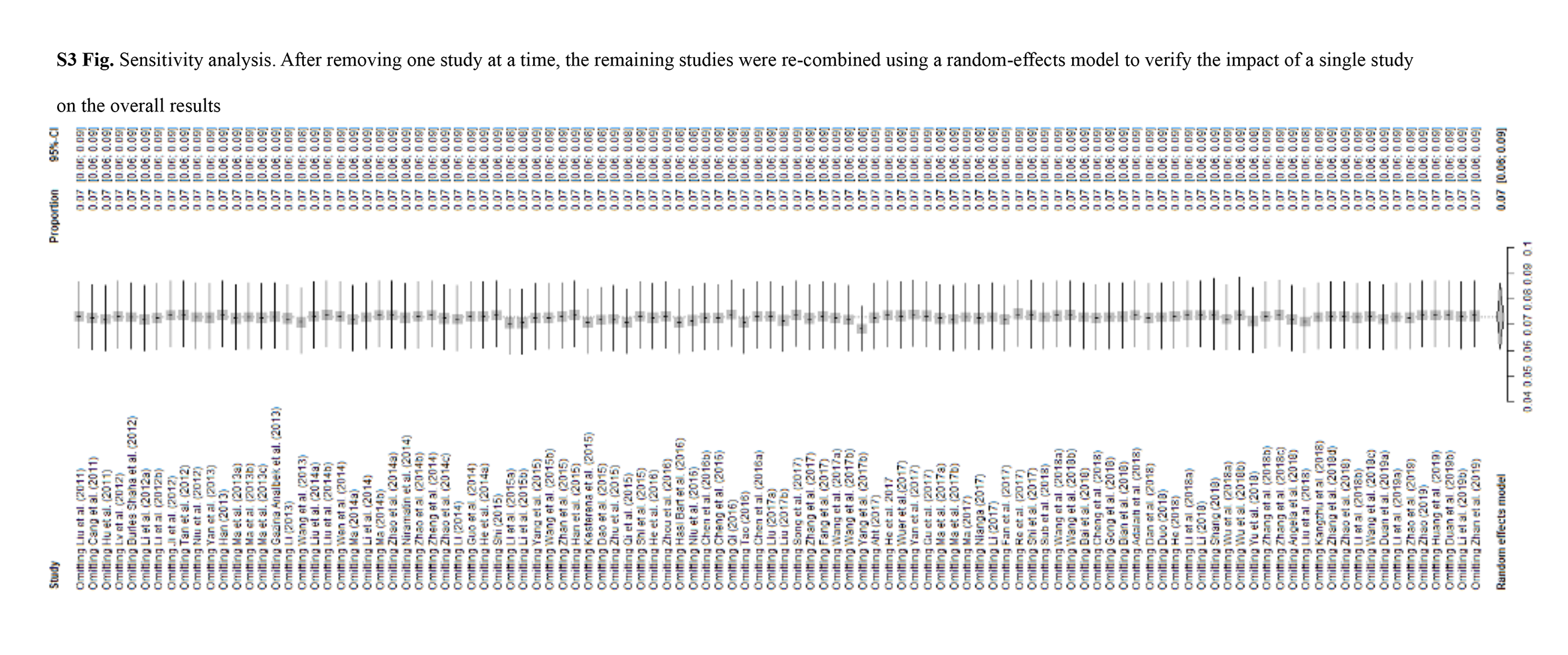

Supplement: S3 Fig — After removing one study at a time, the remaining studies were re-combined using a random-effects model to verify the impact of a single study on the overall results. (TIF) [file pntd.0009268.s003.tif]

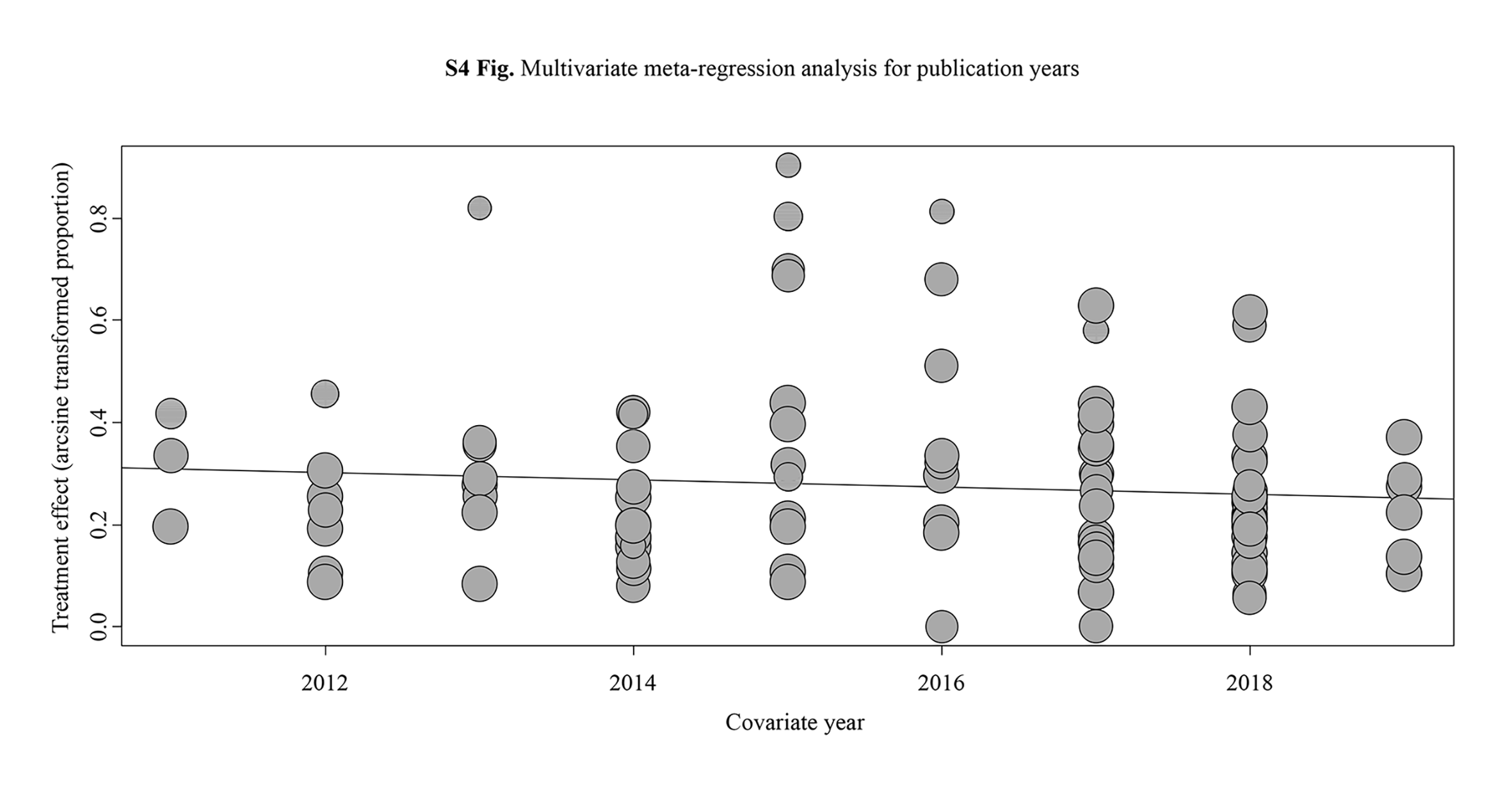

Supplement: S4 Fig — (TIF) [file pntd.0009268.s004.tif]
