# Supplementary material for: Meta-analysis of the prevalence of Echinococcus in dogs in China from 2010 to 2019
Source: PLoS Negl Trop Dis. 2021 Apr 2;15(4):e0009268. doi: 10.1371/journal.pntd.0009268 (PMC8018629; doi:10.1371/journal.pntd.0009268)
Supplement: S2 Table — (DOCX) [file pntd.0009268.s006.docx]

**S2 Table.** Included studies of *Echinococcus* infection of dogs in China.

| Study ID | Sampling time | Province | Detection method | Positive samples/  total samples | Quality score | Quality level |
| --- | --- | --- | --- | --- | --- | --- |
| **Eastern China** |  |  |  |  |  |  |
| Huang et al. (2019) | 2018 | Jiangsu | ELISA | 13/1243 | 3 | Middle |
| **Northern China** |  |  |  |  |  |  |
| Cang et al. (2011) | 2010 | Inner Mongolia | ELISA | 74/682 | 3 | Middle |
| Hu et al. (2011) | 2010 | Inner Mongolia | ELISA | 16/97 | 3 | Middle |
| Li (2013) | 2012.09 | Inner Mongolia | ELISA | 28/226 | 4 | High |
| Li et al. (2018b) | 2016–2017 | Hebei | ELISA | 9/121 | 3 | Middle |
| Liu et al. (2011) | 2010 | Inner Mongolia | ELISA | 356/9405 | 4 | High |
| Shi et al. (2017) | 2012–2014 | Inner Mongolia | ELISA | 647/35752 | 4 | High |
| Wu et al. (2018b) | 2012–2016 | Inner Mongolia | ELISA | 299/13482 | 4 | High |
| Zhang et al. (2018c) | 2014 | Inner Mongolia | ELISA | 1/320 | 3 | Middle |
| **Northwestern China** |  |  |  |  |  |  |
| Adalaiti et al. (2018) | 2015–2017 | Xinjiang | ELISA | 356/34343 | 4 | High |
| Aht (2017) | 2016.10–2017.01 | Gansu | ELISA | 15/180 | 3 | Middle |
| Angela et al. (2018) | 2012–2013 | Ningxia | Multiplex-PCR | 575/3324 | 3 | Middle |
| Burles Shaha et al. (2012) | 2010–2011 | Xinjiang | ELISA | 132/2100 | 3 | Middle |
| Chen et al. (2016a) | 2011 | Xinjiang | ELISA | 74/2219 | 4 | High |
| Chen et al. (2016b) | 2014–2015 | Qinghai | Autopsy and ELISA | 22/220 | 4 | High |
| Cheng et al. (2016) | 2012 | Qinghai | ELISA | 90/838 | 4 | High |
| Cheng et al. (2018) | 2012.06–2012.08 | Qinghai | ELISA | 239/2260 | 4 | High |
| Duan et al. (2019a) | 2017–2018 | Xinjiang | ELISA | 210/1597 | 4 | High |
| Duan et al. (2019b) | 2017 | Ningxia | ELISA | 12/1134 | 2 | Middle |
| Duo (2018) | 2017 | Qinghai | Autopsy and ELISA | 23/435 | 4 | High |
| Fan et al. (2017) | 2010–2015 | Xinjiang | UN | 351/2167 | 1 | Low |
| Fang et al. (2017) | 2011–2015 | Xinjiang | ELISA | 160/5236 | 3 | Middle |
| Gazina Analbek et al. (2013) | 2010–2012 | Xinjiang | ELISA | 181/3690 | 3 | Middle |
| Gu et al. (2017) | ND | Qinghai | ELISA | 21/923 | 3 | Middle |
| Guo et al. (2014) | 2010–2012 | Qinghai | ELISA | 1/41 | 3 | Middle |
| Han (2013) | 2012 | Xinjiang | ELISA | 37/5391 | 5 | High |
| Han et al. (2015) | 2012–2013 | Xinjiang | ELISA | 143/18976 | 5 | High |
| Hasi Bart et al. (2016) | 2015 | Xinjiang | ELISA | 115/292 | 3 | Middle |
| He et al. (2014a) | 2011 | Gansu | ELISA | 73/1902 | 4 | High |
| He et al. (2016) | 2011–2015 | Xinjiang | ELISA | 223/2642 | 3 | Middle |
| Ji et al. (2012) | 2012.01–2012.03 | Xinjiang | ELISA | 10/904 | 5 | High |
| Kesterena et al. (2015) | 2013.04 | Xinjiang | ELISA | 68/164 | 4 | High |
| Li (2014) | 2013.03 | Qinghai | UN | 14/86 | 3 | Middle |
| Li (2017) | 2013–2017 | Gansu | ELISA | 34/620 | 4 | High |
| Li (2018) | 2017 | Gansu | ELISA | 18/1500 | 3 | Middle |
| Li et al. (2012a) | 2010.01 | Qinghai | ICGT | 10/52 | 3 | Middle |
| Li et al. (2012b) | 2011 | Gansu | ELISA | 167/1848 | 5 | High |
| Li et al. (2014) | 2013.03–2013.08 | Xinjiang | ELISA | 71/1134 | 4 | High |
| Li et al. (2015a) | 2014.07 | Qinghai | Autopsy | 21/34 | 2 | Middle |
| Li et al. (2015b) | 2014.10–2014.12 | Qinghai | Flotation method (NaCl) | 31/60 | 2 | Middle |
| Li et al. (2018a) | 2010–2016 | Gansu | ELISA | 136/9211 | 3 | Middle |
| Liu (2017a) | 2015 | Gansu | ELISA | 40/1506 | 4 | High |
| Liu (2017b) | 2015 | Qinghai | Flotation method (NaCl) | 12/40 | 4 | High |
| Liu et al. (2014a) | 2012 | Gansu | ELISA | 117/3001 | 3 | Middle |
| Liu et al. (2014b) | 2012.04–2012.09 | Xinjiang | ELISA | 2/320 | 3 | Middle |
| Liu et al. (2018) | 2012 | Ningxia | Multiplex-PCR | 250/750 | 4 | High |
| Lv et al. (2012) | 2010 | Xinjiang | ELISA | 927/25878 | 3 | Middle |
| Ma (2014a) | 2012 | Qinghai | ELISA | 53/320 | 3 | Middle |
| Ma (2014b) | 2013.05–2013.08 | Qinghai | ELISA | 14/1072 | 4 | High |
| Ma (2017) | 2016.01 | Qinghai | Autopsy and ELISA | 13/187 | 1 | Low |
| Ma et al. (2013a) | 2012 | Xinjiang | ELISA | 39/322 | 4 | High |
| Ma et al. (2013b) | 2011–2013 | Xinjiang | ELISA | 46/720 | 4 | High |
| Ma et al. (2013c) | 2010 | Xinjiang | ELISA | 41/500 | 3 | Middle |
| Ma et al. (2017a) | 2012.06–2012.08 | Qinghai | ELISA | 150/1282 | 4 | High |
| Ma et al.(2017b) | 2012.06–2012.08 | Qinghai | ELISA | 303/2034 | 4 | High |
| Nianga (2017) | 2016–2017 | Qinghai | UN | 69/569 | 2 | Middle |
| Niramatin et al. (2014) | 2013 | Xinjiang | ELISA | 32/440 | 3 | Middle |
| Niu et al. (2012) | 2010 | Gansu | ELISA | 32/621 | 4 | High |
| Qi (2016) | 2015.12–2016.02 | Xinjiang | ELISA | 0/180 | 4 | High |
| Qi et al. (2015) | 2013 | Xinjiang | Autopsy and ELISA | 66/164 | 3 | Middle |
| Re et al. (2017) | 2011–2012 | Xinjiang | ELISA | 0/320 | 3 | Middle |
| Shang (2018) | 2010–2016 | Gansu | ELISA | 3371/77536 | 4 | High |
| Shi (2015) | 2011–2014 | Xinjiang | ELISA | 46/4100 | 4 | High |
| Shi et al. (2015) | 2011–2013 | Xinjiang | ELISA | 92/2433 | 4 | High |
| Song et al. (2017) | 2016 | Xinjiang | ELISA | 18/3970 | 4 | High |
| Tan et al. (2012) | 2011 | Xinjiang | ELISA | 17/2300 | 3 | Middle |
| Tao (2016) | 2015.12 | Qinghai | Flotation method (NaCl) | 19/36 | 4 | High |
| Wang et al. (2013) | 2012.07 | Qinghai | Flotation method (NaCl) | 16/30 | 4 | High |
| Wang et al. (2015b) | 2013–2014 | Xinjiang | ELISA | 5/60 | 3 | Middle |
| Wang et al. (2017a) | 2016 | Xinjiang | ELISA | 63/720 | 4 | High |
| Wang et al. (2017b) | 2012 | Qinghai | ELISA | 231/1295 | 3 | Middle |
| Wang et al. (2018a) | 2015 | Gansu | ELISA | 115/5654 | 4 | High |
| Wen et al. (2014) | 2010–2013 | Xinjiang | ELISA | 95/3280 | 4 | High |
| Wu et al. (2018a) | 2015–2017 | Qinghai | Autopsy and ELISA | 62/458 | 3 | Middle |
| Wu et al. (2018b) | 2012–2016 | Shaanxi | ELISA | 0/640 | 4 | High |
| Wu et al. (2018b) | 2012–2016 | Gansu | ELISA | 1133/23056 | 4 | High |
| Wu et al. (2018b) | 2012–2016 | Ningxia | ELISA | 195/6445 | 4 | High |
| Wu et al. (2018b) | 2012–2016 | Qinghai | ELISA | 1505/11563 | 4 | High |
| Wu et al. (2018b) | 2012–2016 | Xinjiang | ELISA | 649/32055 | 4 | High |
| Wuer et al. (2017) | 2012.08–2013.09 | Xinjiang | ELISA | 223/8493 | 5 | High |
| Yan et al. (2013) | 2012 | Xinjiang | ELISA | 83/1100 | 3 | Middle |
| Yang et al. (2015) | 2011–2013 | Xinjiang | ELISA | 71/730 | 4 | High |
| Yu et al. (2018) | 2016–2017 | Xinjiang | ELISA | 83/270 | 4 | High |
| Zhan et al. (2019) | 2016 | Gansu | ELISA | 73/3923 | 4 | High |
| Zhan et al. (2015) | 2011–2013 | Gansu | ELISA | 55/1255 | 3 | Middle |
| Zhang et al. (2017) | 2016 | Qinghai | Flotation method (NaCl) | 5/31 | 3 | Middle |
| Zhang et al. (2018b) | 2016 | Gansu | ELISA | 76/2520 | 4 | High |
| Zhang et al. (2018d) | 2017.05–2017.10 | Qinghai | ELISA | 5/180 | 3 | Middle |
| Zhao (2019) | 2017 | Gansu | ELISA | 2/180 | 3 | Middle |
| Zhao et al. (2014a) | 2012.08–2013.09 | Xinjiang | ELISA | 719/29874 | 4 | High |
| Zhao et al. (2014b) | 2012 | Ningxia | Autopsy and ELISA | 237/7838 | 4 | High |
| Zhao et al. (2014c) | 2012 | Qinghai | ELISA | 38/320 | 4 | High |
| Zhao et al. (2018) | 2016–2017 | Qinghai | Autopsy and ELISA | 5/186 | 3 | Middle |
| Zheng et al. (2014) | 2012.03–2012.10 | Ningxia | ELISA | 5/320 | 4 | High |
| Zhou et al. (2016) | 2015.06–2015.12 | Ningxia | ELISA | 187/4620 | 5 | High |
| Zhu et al. (2015) | 2010–2012 | Gansu | ELISA | 1115/7500 | 5 | High |
| **Southwestern China** |  |  |  |  |  |  |
| Bai et al. (2018) | 2016.08–2016.10 | Tibet | ELISA | 64/932 | 3 | Middle |
| Bian et al. (2018) | 2016.08–2016.10 | Tibet | ELISA | 92/1946 | 4 | High |
| Dan et al. (2018) | 2016.08–2016.10 | Tibet | ELISA | 109/1081 | 4 | High |
| Dao et al. (2015) | 2011 | Sichuan | ELISA | 3020/16825 | 3 | Middle |
| Gong et al. (2018) | 2016.08–2016.10 | Tibet | ELISA | 78/1358 | 3 | Middle |
| He (2018) | 2015.03–2016.03 | Sichuan | ELISA | 45/1196 | 5 | High |
| He et al. (2017) | 2015 | Sichuan | ELISA | 8/554 | 4 | High |
| Kangzhu et al. (2018) | 2016.08–2016.11 | Tibet | ELISA | 458/7259 | 5 | High |
| Li et al. (2019a) | 2016 | Tibet | ELISA | 552/7564 | 4 | High |
| Li et al. (2019b) | 2012–2017 | Yunnan | ELISA | 965/19812 | 4 | High |
| Niu et al. (2016) | 2015.05 | Tibet | ELISA | 54/226 | 5 | High |
| Sub et al. (2018) | 2016.08–2016.12 | Tibet | ELISA | 66/1047 | 4 | High |
| Wang et al. (2018c) | 2016.08–2016.10 | Tibet | ELISA | 18/501 | 4 | High |
| Wang et al. (2018b) | 2016.10–2017.10 | Yunnan | ELISA | 1/251 | 3 | Middle |
| Wu et al. (2018b) | 2012–2016 | Sichuan | ELISA | 331/11200 | 4 | High |
| Wu et al. (2018b) | 2012–2016 | Tibet | ELISA | 552/7564 | 4 | High |
| Wu et al. (2018b) | 2012–2016 | Yunnan | ELISA | 86/5827 | 4 | High |
| Yan et al. (2017) | 2017.05–2017.10 | Yunnan | ELISA | 0/104 | 3 | Middle |
| Yang et al. (2017b) | 2015 | Sichuan | ELISA | 9192/26653 | 3 | Middle |
| Zhao et al. (2019) | 2016–2018 | Yunnan | ELISA | 35/436 | 4 | High |
