# Supplementary material for: Meta-analysis of the prevalence of Echinococcus in dogs in China from 2010 to 2019
Source: PLoS Negl Trop Dis. 2021 Apr 2;15(4):e0009268. doi: 10.1371/journal.pntd.0009268 (PMC8018629; doi:10.1371/journal.pntd.0009268)
Supplement: S3 Table — (DOCX) [file pntd.0009268.s007.docx]

**S3 Table.** The quality scores and the literature list in this meta-analysis.

|  | **Reference ID** | **No. tested** | **No. positive** | **Prevalence** | **Study design** | **Random sampling or not** | **Sampled method clearly or not** | **Sampled method detailedly or not** | **The number of samples is ≥ 200 or not** | **Four or more risk factors or not** | **Score** | **Study Quality** |
| --- | --- | --- | --- | --- | --- | --- | --- | --- | --- | --- | --- | --- |
| 1 | Duan et al.(2019a) | 1597 | 210 | 0.131 | Cross sectional | Y | Y | N | Y | Y | 4 | high |
| 2 | Liu (2017a) | 1506 | 40 | 0.027 | Cross sectional | Y | Y | N | Y | Y | 4 | high |
| 3 | Liu (2017b) | 40 | 12 | 0.300 | Cross sectional | Y | Y | Y | N | Y | 4 | high |
| 4 | Song et al.(2017) | 3970 | 18 | 0.005 | Cross sectional | Y | Y | N | Y | Y | 4 | high |
| 5 | Zhang et al.(2017) | 31 | 5 | 0.161 | Cross sectional | N | Y | Y | N | Y | 3 | middle |
| 6 | Fang et al.(2017) | 5236 | 160 | 0.031 | Cross sectional | N | Y | N | Y | Y | 3 | middle |
| 7 | Wang et al.(2017a) | 720 | 63 | 0.088 | Cross sectional | N | Y | Y | Y | Y | 4 | high |
| 8 | Wang et al.(2017b) | 1295 | 231 | 0.178 | Cross sectional | N | Y | N | Y | Y | 3 | middle |
| 9 | Yang et al.(2017b) | 26653 | 9192 | 0.345 | Cross sectional | N | Y | N | Y | Y | 3 | middle |
| 10 | Aht (2017) | 180 | 15 | 0.083 | Cross sectional | N | Y | Y | N | Y | 3 | middle |
| 11 | Liu et al.(2011) | 9405 | 356 | 0.038 | Cross sectional | Y | Y | N | Y | Y | 4 | high |
| 12 | Lv et al.(2012) | 25878 | 927 | 0.036 | Cross sectional | N | Y | N | Y | Y | 3 | middle |
| 13 | Burles Shaha et al.(2012) | 2100 | 132 | 0.063 | Cross sectional | N | Y | N | Y | Y | 3 | middle |
| 14 | Cang et al.(2011) | 682 | 74 | 0.109 | Cross sectional | N | Y | N | Y | Y | 3 | middle |
| 15 | Shi (2015) | 4100 | 46 | 0.011 | Cross sectional | Y | Y | N | Y | Y | 4 | high |
| 16 | Li et al.(2015a) | 34 | 21 | 0.618 | Cross sectional | N | Y | N | N | Y | 2 | middle |
| 17 | Li et al.(2015b) | 60 | 31 | 0.517 | Cross sectional | N | Y | N | N | Y | 2 | middle |
| 18 | Yang et al.(2015) | 730 | 71 | 0.097 | Cross sectional | Y | Y | N | Y | Y | 4 | high |
| 19 | Wang et al.(2015b) | 60 | 5 | 0.083 | Cross sectional | Y | Y | N | N | Y | 3 | middle |
| 20 | He et al.(2016) | 2642 | 223 | 0.084 | Cross sectional | N | Y | N | Y | Y | 3 | middle |
| 21 | Zhou et al.(2016) | 4620 | 187 | 0.040 | Cross sectional | Y | Y | Y | Y | Y | 5 | high |
| 22 | Hasi Bart et al.(2016) | 292 | 115 | 0.394 | Cross sectional | N | Y | N | Y | Y | 3 | middle |
| 23 | Zhan et al.(2015) | 1255 | 55 | 0.044 | Cross sectional | N | Y | N | Y | Y | 3 | middle |
| 24 | Han et al.(2015) | 18976 | 143 | 0.008 | Cross sectional | Y | Y | Y | Y | Y | 5 | high |
| 25 | Niu et al.(2016) | 226 | 54 | 0.239 | Cross sectional | Y | Y | Y | Y | Y | 5 | high |
| 26 | Li et al.(2012a) | 52 | 10 | 0.192 | Cross sectional | Y | Y | N | N | Y | 3 | middle |
| 27 | Li et al.(2012b) | 1848 | 167 | 0.090 | Cross sectional | Y | Y | Y | Y | Y | 5 | high |
| 28 | Ji et al.(2012) | 904 | 10 | 0.011 | Cross sectional | Y | Y | Y | Y | Y | 5 | high |
| 29 | Tan et al.(2012) | 2300 | 17 | 0.007 | Cross sectional | N | Y | N | Y | Y | 3 | middle |
| 30 | Chen et al.(2016b) | 220 | 22 | 0.100 | Cross sectional | Y | Y | N | Y | Y | 4 | high |
| 31 | Cheng et al.(2016) | 838 | 90 | 0.107 | Cross sectional | Y | Y | N | Y | Y | 4 | high |
| 32 | He et al.2017 | 554 | 8 | 0.014 | Cross sectional | Y | Y | N | Y | Y | 4 | high |
| 33 | Wuer et al.2017 | 8493 | 223 | 0.026 | Cross sectional | Y | Y | Y | Y | Y | 5 | high |
| 34 | Qi (2016) | 180 | 0 | 0.000 | Cross sectional | Y | Y | Y | N | Y | 4 | high |
| 35 | Tao (2016) | 36 | 19 | 0.528 | Cross sectional | Y | Y | Y | N | Y | 4 | high |
| 36 | Yan et al.2017 | 104 | 0 | 0.000 | Cross sectional | Y | Y | N | N | Y | 3 | middle |
| 37 | Sub et al.(2018) | 1047 | 66 | 0.063 | Cross sectional | Y | Y | N | Y | Y | 4 | high |
| 38 | Wang et al.(2018a) | 5654 | 115 | 0.020 | Cross sectional | Y | Y | N | Y | Y | 4 | high |
| 39 | Wang et al.(2018b) | 251 | 1 | 0.004 | Cross sectional | N | Y | N | Y | Y | 3 | middle |
| 40 | Bai et al.(2018) | 932 | 64 | 0.069 | Cross sectional | N | Y | N | Y | Y | 3 | middle |
| 41 | Cheng et al.(2018) | 2260 | 239 | 0.106 | Cross sectional | Y | Y | N | Y | Y | 4 | high |
| 42 | Gong et al.(2018) | 1358 | 78 | 0.057 | Cross sectional | N | Y | N | Y | Y | 3 | middle |
| 43 | Bian et al.(2018) | 1946 | 92 | 0.047 | Cross sectional | Y | Y | N | Y | Y | 4 | high |
| 44 | Adalaiti et al.(2018) | 34343 | 356 | 0.010 | Cross sectional | Y | Y | N | Y | Y | 4 | high |
| 45 | Li et al.(2019a) | 7564 | 552 | 0.073 | Cross sectional | Y | Y | N | Y | Y | 4 | high |
| 46 | Dan et al.(2018) | 1081 | 109 | 0.101 | Cross sectional | Y | Y | N | Y | Y | 4 | high |
| 47 | Duo (2018) | 435 | 23 | 0.053 | Cross sectional | N | Y | Y | Y | Y | 4 | high |
| 48 | Gu et al.(2017) | 923 | 21 | 0.023 | Cross sectional | N | Y | N | Y | Y | 3 | middle |
| 49 | He (2018) | 1196 | 45 | 0.038 | Cross sectional | Y | Y | Y | Y | Y | 5 | high |
| 50 | Li et al.(2018a) | 9211 | 136 | 0.015 | Cross sectional | N | Y | N | Y | Y | 3 | middle |
| 51 | Li (2018) | 1500 | 18 | 0.012 | Cross sectional | N | Y | N | Y | Y | 3 | middle |
| 52 | Ma et al.(2017a) | 1282 | 150 | 0.117 | Cross sectional | Y | Y | N | Y | Y | 4 | high |
| 53 | Ma et al.(2017b) | 2034 | 303 | 0.149 | Cross sectional | Y | Y | N | Y | Y | 4 | high |
| 54 | Ma (2017) | 187 | 13 | 0.070 | Cross sectional | N | Y | N | N | N | 1 | low |
| 55 | Shang (2018) | 77536 | 3371 | 0.043 | Cross sectional | Y | Y | N | Y | Y | 4 | high |
| 56 | Wu et al.(2018a) | 458 | 62 | 0.135 | Cross sectional | N | Y | N | Y | Y | 3 | middle |
| 57 | Wu et al.(2018b) | 111832 | 4750 | 0.042 | Cross sectional | Y | Y | N | Y | Y | 4 | high |
| 58 | Yu et al.(2018) | 270 | 83 | 0.307 | Cross sectional | Y | Y | N | Y | Y | 4 | high |
| 59 | Zhang et al.(2018b) | 2520 | 76 | 0.030 | Cross sectional | Y | Y | N | Y | Y | 4 | high |
| 60 | Zhang et al.(2018c) | 320 | 1 | 0.003 | Cross sectional | N | Y | N | Y | Y | 3 | middle |
| 61 | Angela et al.(2018) | 3324 | 575 | 0.173 | Cross sectional | N | Y | N | Y | Y | 3 | middle |
| 62 | Liu et al.(2018) | 750 | 250 | 0.333 | Cross sectional | N | Y | Y | Y | Y | 4 | high |
| 63 | Liu et al.(2014a) | 3001 | 117 | 0.039 | Cross sectional | N | Y | N | Y | Y | 3 | middle |
| 64 | Liu et al.(2014b) | 320 | 2 | 0.006 | Cross sectional | N | Y | N | Y | Y | 3 | middle |
| 65 | Wen et al.(2014) | 3280 | 95 | 0.029 | Cross sectional | Y | Y | N | Y | Y | 4 | high |
| 66 | Yan et al.(2013) | 1100 | 83 | 0.075 | Cross sectional | N | Y | N | Y | Y | 3 | middle |
| 67 | Han (2013) | 5391 | 37 | 0.007 | Cross sectional | Y | Y | Y | Y | Y | 5 | high |
| 68 | Ma et al.(2013a) | 322 | 39 | 0.121 | Cross sectional | N | Y | Y | Y | Y | 4 | high |
| 69 | Ma et al.(2013b) | 720 | 46 | 0.064 | Cross sectional | Y | Y | N | Y | Y | 4 | high |
| 70 | Ma et al.(2013c) | 500 | 41 | 0.082 | Cross sectional | N | Y | N | Y | Y | 3 | middle |
| 71 | Ma (2014a) | 320 | 53 | 0.166 | Cross sectional | N | Y | N | Y | Y | 3 | middle |
| 72 | Li et al.(2014) | 1134 | 71 | 0.063 | Cross sectional | Y | Y | N | Y | Y | 4 | high |
| 73 | Ma (2014b) | 1072 | 14 | 0.013 | Cross sectional | Y | Y | N | Y | Y | 4 | high |
| 74 | Zhao et al.(2014a) | 29874 | 719 | 0.024 | Cross sectional | N | Y | Y | Y | Y | 4 | high |
| 75 | Niramatin et al.(2014) | 440 | 32 | 0.073 | Cross sectional | N | Y | N | Y | Y | 3 | middle |
| 76 | Zhao et al.(2014b) | 7838 | 237 | 0.030 | Cross sectional | Y | Y | N | Y | Y | 4 | high |
| 77 | Zheng et al.(2014) | 320 | 5 | 0.016 | Cross sectional | Y | Y | N | Y | Y | 4 | high |
| 78 | Zhao et al.(2014c) | 320 | 38 | 0.119 | Cross sectional | Y | Y | N | Y | Y | 4 | high |
| 79 | Kesterena et al.(2015) | 164 | 68 | 0.415 | Cross sectional | Y | Y | Y | N | Y | 4 | high |
| 80 | Dao et al.(2015) | 16825 | 3020 | 0.179 | Cross sectional | Y | Y | N | Y | N | 3 | middle |
| 81 | Gazina Analbek et al.(2013) | 3690 | 181 | 0.049 | Cross sectional | N | Y | N | Y | Y | 3 | middle |
| 82 | Nianga (2017) | 569 | 69 | 0.121 | Cross sectional | Y | N | N | Y | N | 2 | middle |
| 83 | Kangzhu et al.(2018) | 7259 | 458 | 0.063 | Cross sectional | Y | Y | Y | Y | Y | 5 | high |
| 84 | Zhang et al.(2018d) | 180 | 5 | 0.028 | Cross sectional | Y | Y | N | N | Y | 3 | middle |
| 85 | Zhu et al.(2015) | 7500 | 1115 | 0.149 | Cross sectional | Y | Y | Y | Y | Y | 5 | high |
| 86 | li (2014) | 86 | 14 | 0.163 | Cross sectional | Y | M | Y | N | Y | 3 | middle |
| 87 | Guo et al.(2014) | 41 | 1 | 0.024 | Cross sectional | Y | Y | N | N | Y | 3 | middle |
| 88 | Zhao et al.(2018) | 186 | 5 | 0.027 | Cross sectional | Y | Y | N | N | Y | 3 | middle |
| 89 | Zhao et al.(2019) | 436 | 35 | 0.080 | Cross sectional | Y | Y | N | Y | Y | 4 | high |
| 90 | Zhao (2019) | 180 | 2 | 0.011 | Cross sectional | N | Y | Y | N | Y | 3 | middle |
| 91 | Chen et al.(2016a) | 2219 | 74 | 0.033 | Cross sectional | Y | Y | N | Y | Y | 4 | high |
| 92 | Huang et al.(2019) | 1243 | 13 | 0.010 | Cross sectional | N | Y | N | Y | Y | 3 | middle |
| 93 | Qi et al.(2015) | 164 | 66 | 0.402 | Cross sectional | Y | Y | N | N | Y | 3 | middle |
| 94 | Li (2013) | 226 | 28 | 0.124 | Cross sectional | N | Y | Y | Y | Y | 4 | high |
| 95 | Niu et al.(2012) | 621 | 32 | 0.052 | Cross sectional | N | Y | Y | Y | Y | 4 | high |
| 96 | Wang et al.(2013) | 30 | 16 | 0.533 | Cross sectional | Y | Y | Y | N | Y | 4 | high |
| 97 | Li et al.(2018b) | 121 | 9 | 0.074 | Cross sectional | Y | Y | N | N | Y | 3 | middle |
| 98 | Duan et al.(2019b) | 1134 | 12 | 0.011 | Cross sectional | N | Y | N | Y | N | 2 | middle |
| 99 | Li et al.(2019b) | 19812 | 965 | 0.049 | Cross sectional | Y | Y | N | Y | Y | 4 | high |
| 100 | Li (2017) | 620 | 34 | 0.055 | Cross sectional | Y | Y | N | Y | Y | 4 | high |
| 101 | Fan et al.(2017) | 2167 | 351 | 0.162 | Cross sectional | N | N | N | Y | N | 1 | low |
| 102 | Re et al.(2017) | 320 | 0 | 0.000 | Cross sectional | N | Y | N | Y | Y | 3 | middle |
| 103 | Wang et al.(2018c) | 501 | 18 | 0.036 | Cross sectional | Y | Y | N | Y | Y | 4 | high |
| 104 | Shi et al (2015) | 2433 | 92 | 0.038 | Cross sectional | Y | Y | N | Y | Y | 4 | high |
| 105 | Zhan et al (2019) | 3923 | 73 | 0.019 | Cross sectional | N | Y | Y | Y | Y | 4 | high |
| 106 | He et al (2014a) | 1902 | 73 | 0.038 | Cross sectional | Y | Y | N | Y | Y | 4 | high |
| 107 | Hu et al (2011) | 97 | 16 | 0.165 | Cross sectional | N | Y | Y | N | Y | 3 | middle |
| 108 | Shi et al (2017) | 35752 | 647 | 0.018 | Cross sectional | N | Y | Y | Y | Y | 4 | high |

Y*: Yes; N*: No.

**References**

1. Duan, Z.Z., Li, J., Yang, S.Z., Bai, Z.H., He, Z.L., 2019.Investigation and analysis on the prevalence of animal hydatid disease in Aksu region of Xinjiang in 2017-2018. Grass-Feeding Livestock 2019, 39-42.
2. Liu, X.M., 2015.Prevalence of hydatid disease in Ganzhou District in 2015.Foreign Medical Sciences 37, 35-37.
3. Liu, Y.M., 2017.Investigation on the infection of Echinococcus granulosus in Guide County. Shandong Journal of Animal Science and Veterinary Medicine 39,49-50.
4. Song, F., Li, H.J., Liao, X.F., 2017.The ananlysis of the screening results of hydatidosis in key Areas of Urumqi in 2016.Journal of Diseases Monitor & Control 11,859-860.
5. Zhang, H.C., Zhang, Y.Q., 2017.Investigation on the infection of hydatid disease in dogs and sheep in Zhugu Township, Menyuan County.Chinese Qinghai Journal of Animal and Veterinary Sciences.47,46-47.
6. Fang, J., Yang, H.D., Xu, J., Wang, J., 2017.Epidemiology and prevention of echinococcosis in Kuitun District from 2011 to 2015.Chinese Journal of Health Laboratory Technology.27,1044-1045+1049.
7. Wang, W.X., Li.Y.T., Wang, T., LI, A.Q., Cai, K.J., Xu, M., 2017.Epidemiological investigation report on echinococcosis in Urumqi in 2016.Xinjiang Animal Husbandry.39,368-370.
8. Wang, Y.S., Ma, X., Han, X.M., Zhang, J.X., Liu, Y.F., Cai.H.X., Lei.W., Wang, W., Liu, J., Zhang, J.N., Liu, P.Y., Zhang, X.F., 2017.Epidemiological survey of hydatid disease in Haibei Tibetan Autonomous Prefecture, Qinghai Province. Journal of Pathogen Biology.12,441-443.
9. Yang, A.G., Guo, L., Mao, G.Q., Hou, w., Wen, H., Chen. D., Ying, L.J., Mo, Q., Li, Q., Zhang, Z.H., Yan, D.B., Lu, Z.P., 2017.Sichuan: Do more measures and improve the model. China Animal Industry.2017,26-28.
10. A, h.t., 2017.Epidemiological survey of echinococcosis in Akesai Kazakh Autonomous County. China Animal Health.19,80-81.
11. Liu, X.M., Tian, F.Y., Zheng,G.J., Wang,H., 2011.A Survey on epidemic status of echinococcosis in Xi Lin Guo Le Meng,Inner Mongolia.Journal of Diseases Monitor & Control 5,721-3.
12. Lv,J.Z., Lin,D.H., Li,G.L.,2012.Investigation on the status of prevention and control of echinococcosis in Changji Hui Autonomous Prefecture, Xinjiang, 2008-2011.Bulletin of Disease Control & Prevention(China)27,26-7.
13. Burles Shaha., Xue,F., Heiner Torregen.,2012.Analysis on prevention and treatment of echinococcosis in Toli County, Xinjiang.Bulletin of Disease Control & Prevention(China) 27.57-8.
14. Cang,J.H., Song,A.J., Chen,W., Yang,D., Zhao,S.H., Liu,X.S., 2011.Investigation report on the infection status of Echinococcus sinensis in the border area of Inner Mongolia.Animal Husbandry and Feed Science 32,117-20
15. Shi,X.J., 2015. Study on the epidemic characteristics of hydatidosis and the related influencing factors.China Rural Health 8,44-45.
16. Li,G.P., 2015. Investigation of Echinococcus infection in Qinghai Province and detection of its comprehensive control effect.China Cattle Science 41,12-14.
17. Li,S.F., 2015. A survey of echinococcosis in dogs and cattle and sheep in Minhe County.Chinese Qinghai Journal of Animal and Veterinary Sciences 45,33.
18. Yang,F., 2015. Survey of echinococcosis infection in domestic animals in Wensu County, Xinjiang.Grass-Feeding Livestock 2,35-39+48.
19. Wang,W.,Investigation of Echinococcus granulosus infection in dogs in some areas of Shihezi.Xinjiang Farm Research of Science and Technology 38,31-33.
20. He, L.X., Wang, B., Chen, Z.Y., Li, S., Qiu, G.B., Wu, S., Alidingba, Yang, H.K., 2019.Investigation on Prevention and Control Effect of Livestock Hydatid Disease in Zhaosu Reclamation Area of Yili, Xinjiang. China Animal Health Inspection 2016, 33, 12-4.
21. Zhou, H.N., Wang, J.X., Zhang, W., Wang, Y.M., Yan, X.Q., Li, P., Wang, X.L., 2016.An Epidemiological Survey on Livestock Hydatid Disease in Ningxia in 2015. China Animal Health Inspection 2016, 33, 11-3+80.
22. Hasi Bart, Song, Y.C., Battelle, Hu La, Li, L., 2016.Investigation on canine hydatidosis infection in Turhut shepherd dogs in pastoral areas of Bazhou and Jingxian, Xinjiang. Modern Animal Husbandry, 2016, 64.
23. Zhan, F., Shi, W.G., Liu, Q.Y., Liu, X., Pan, Y.H.,2015. Investigation on Infection of Livestock Hydatidosis in Some Areas of Gansu Province. Journal of Traditional Chinese Veterinary Medicine, 2015, 34, 73-5.
24. Han, F., Wang, B.Q., Li, F.K., Song, J.Y., 2015. Investigation on Echinococcus granulosus Infection in Domestic Dogs of Corps from 2011 to 2013. Modern Preventive Medicine, 2015, 42, 906-7+911.
25. Niu, Y.L., Wu, W.P., Guan, Y.Y., Wang, L.Y., Han, S., Gongsang, Q.Z., Gang, Z., Ciren, W.D., Yixi, D.Z., Ciren, Q.Z., Cesang, C.R., Jimi, Q.Z., 2016. Dog Fecal Contamination by Echinococcus in Cuomei County of Tibet in 2015. Chinese Journal of Parasitology and Parasitic Diseases, 2016, 34,137-143.
26. Li, W., Fu, Y., Duo, H., 2012. Investigation on the infection of hydatid disease before and after deworming in the state-owned ranch of Yushu County. Chinese Qinghai Journal of Animal and Veterinary Sciences 42,40.
27. Li, H, J., Niu, W. D., 2012. Epidemiological investigation and analysis of echinococcosis in Sunan County, Gansu Province. Bulletin of Disease Control & Prevention (China) 27,24-5.
28. Ji, W.L., Zhang, J.H., 2012 The prevalence survey of hydatid disease in Midong district of Rumqi city in 2012. Chinese Journal of Health Laboratory Technology. 22,2194-5.
29. Tan, H., Lin, C.B., Dong, S.W., Zhang, Q., Duan., S.L., Duan, L.J., 2012 In 2011, Xinjiang Production and Construction Corps Investigation on the prevalence of echinococcosis. Bulletin of Disease Control &Prevention (China). 27,70-1.
30. Chen, C.J., Fan, X.L., Wang, G.C., 2016.Investigation of Echinococcosis epidemic situation in Huangyuan County of Qinghai Province. Journal of Traditional Chinese Veterinary Medicine 2016, 42-43
31. Cheng, S.L., Wang, H., Ma, X., Zhang, J.X., Liu, Y.F., Cai, H.X., Liu, P.X., Ma, J.Y., He, D.L., Wu, X.H., Han, X.M., Wang, Y.S., Liu, H.Q., Zhao, Y.M., Liu, B.R., Zeng, C., Wang, W., Du, R., Lei, W., Su, G.M., Zhou, B.J., Song, C.X., Wang, Y.J., 2016.An Epidemiological Survey on Echinococcosis in Yushu Prefecture of Qinghai Province. Chinese Journal of Parasitology and Parasitic Diseases 34,547-551
32. He, W., Shang, J.Y., Yu, W.J., Zhang, G.J., Wang, Q., Huang, Y., Zhong, B., Zhang, Q., 2017.Study on the Current Status Investigation of Epidemiology of Echinococcosis (Hydatidosis) in Shiqu County of Sichuan Province. Journal of Preventive Medicine Information 33, 850-854
33. Wuer, M., Usman, I., Simayi, A., Hou, Y.Y., Xiao, N., 2017.A survey on Echinococcus infections in animals in Xinjiang Uygur Autonomous Region. Chinese Journal of Parasitology and Parasitic Diseases 35, 145-149
34. Qi, Q.F., 2016.Epidemiological investigation of human and animal echinococcosis in Toxon County. Contemporary Animal Husbandry 2016, 40-41
35. Tao, L.D., 2016.Investigation on canine Echinococcus granulosus infection in Gonghe County. Shandong Journal of Animal Science and Veterinary Medicine 37, 65-66
36. Yan, S.L., He, S.W., 2017.Epidemiological Investigation of Livestock Hydatidosis in Yongsheng County. Veterinary Orientation 2017, 151-152
37. Sub, R.L., Zheng, Y.L., Dan, J.W.J., Long, Z.Y., Dan, Z.Q.Z., Ai, J.J., Wang, D.M., Li, J.Z., 2018. Epidemiological status of echinococcosis in Lhasa. Chinese Journal of Parasitology and Parasitic Diseases 36, 58-62, 74.
38. Wang, D., Feng, Y., Li, F., Liu, Z.J., Liang, H., Yang, G.B., Ge, P.F., 2018. Investigation on epidemic status of echinococcosis in Gansu Province in 2015. Bulletin of Disease Control & Prevention (China) 33, 13-6.
39. Wang, J.Z., Yin, S.Q., Yan, H., Tang, Z.Y., Li, X.S., Liu, A.H., 2018. Investigation on clues of echinococcosis in Tengchong City, Yunnan Province. Journal of Tropical Diseases and Parasitology 16, 38-9.
40. Bai, M.Y.J, Wu, W.P., He, R.F., Gong, S.Q.Z., Kang, Z.Y.X., Suo, L.W.J., Li, B., 2018. Investigation on the current situation of echinococcosis in Shannan City. Chinese Journal of Parasitology and Parasitic Diseases 36, 63-7.
41. Cheng, S.L., Wang, H., Ma, X., Zhang, J.X., Cai, H.X., Wang, Y.S., Liu, Y.F., Ma, J.Y., Liu, P.Y., Lei, W., 2018. Epidemiological survey of echinococcosis in Xining City, Qinghai Province. Chinese Journal of Public Health 34, 700-2.
42. Gong, S.Q.Z., Li, B., Chen, W.Q., Ga, S., Suo, L.W.J., Wang, D.M., Kang, Z.Y.X., Li, J.Z., 2018, Analysis of the current situation of echinococcosis in Changdu City. Chinese Journal of Parasitology and Parasitic Diseases 36, 68-74.
43. Bian, B.Z.M., Li, B., Chen, W.Q., Wang, D.M., Xiao, D., Bian, B., Gong, S.Q.Z., 2018. Analysis of the current situation of echinococcosis in Xigaze City. Chinese Journal of Parasitology and Parasitic Diseases 36, 80-6.
44. Adalaiti, H., Mangkuli, H., Ahiri, R.K., Halinur, G., Ajguri, R., Wang, Q.Y., Dang, X.L., Abreti, A., 2018. An investigation on the current situation of echinococcosis in Tacheng, Xinjiang. Chinese Journal of Parasitology and Parasitic Diseases 36, 565-70.
45. Li, B., Quzhen, G., Xue, C.Z., Han, S., Chen, W.Q., Yan, X.L., Li, Z.J., Quick, M.L., Huang, Y., Xiao, N., Wang, Y., Wang, L.Y., Zuoga, G., Bianba., Gangzhu., Ma, B.C., Gasong., Wei, X.G., Niji., Zheng, C.J., Wu, W.P., Zhou, X.N., 2019. Epidemiological survey of echinococcosis in Tibet Autonomous Region of China. Infect Dis Poverty 28, 8, 29.
46. Dan, Z.W.J., Xue, C.Z., Ai, J.J., Gong, S.Q.Z., Luo, Z.H., Dan, Z.Q.Z., WeiX.G., Zheng, C.J., 2018. Analysis of echinococcosis prevalence in Nagqu Prefecture. Chinese Journal of Parasitology and Parasitic Diseases,36,1
47. Duo, J., 2018. Investigation of Echinococcus canis infection in Hainan. Shanghai Journal of Animal Husbandry and Veterinary Medicine,3
48. Gu, D.H., Yang, J.Y., Li, G.P., Yin, P.C., Ha, L.G., Zhang, C.D.Z.M., Du, M.Z., 2017.An investigation of tapeworm infection in dogs.Shandong Journal of Animal Science and Veterinary Medicine,11
49. He, W., Shang, J.Y., Chen, F., Wang, S.Y., Wang, Q., Huang, Y., Wang, Q., Zhong, B., Zhang, Q., 2018. Regularity of Canine Echinococcosis in Epidemic Areas in Sichuan. Journal of Preventive Medicine Information34,2
50. Li, D.H., Guo, Q.S., Shang, W.J., 2018. Epidemiological characteristics of echinococcosis in Zhuoni County Gansu Province. Chinese Journal of Endemiology,37,8
51. Li, Q.H., 2018.Detection and analysis of hydatid antigen in dog feces in Zhuoni County.China Health Care & Nutrition,4
52. Ma, X., Wang, H., Cheng, S.L., Zhang, J.X., Wang, Y.S., Liu, Y.F., Ma, J.Y., Cai, H.X., Liu, P.Y., Han, X.M., Liu, H.Q., Zhao, Y.M., Wang, W., Lei, W., Du, R., Su, G.M., Liu, N., Shi, K.M., Zhang, X.Y., 2017. Epidemiological investigation on echinococcosis in Huangnan Tibetan Autonomous Prefecture of Qinghai Province.Chinese Journal of Parasitology and Pa rasitic Diseases. 35,5
53. Ma, X., Wang, H., Zhang, J.X., Wang, Y.S., Cheng, S.L., Liu, Y.F., Ma, J.Y., Cai, H.X., Liu, P.Y., Han, X.M., Liu, H.Q., Zhao, Y.M., Wang, W., Du, R., Lei, W., Su, G.M., Zhao, C.Z., Gang, J., Gang, Z., Qiu, G., 2017.Epidemiological investigation on hydatid disease/echinococcosis in Guoluo Tibetan Autonomous Prefecture in Qinghai Province.Chinese Journal of Parasitology and Pa rasitic Disease.35,4
54. Ma, Z.X., Tan, M.L., 2017. Investigation and suggestion on the control of canine hydatidosis in Huangyuan County.Chinese Journal of Traditional Veterinary Science.3
55. Shang, W.J., Zhang, S.W., 2018. Effective analysis of prevention and control of echinococcosis in Gannan Tibetan autonomous prefecture.Bulletin of Disease Control & Prevention(China),33,1
56. Wu, Y.C.Q.K., Yuan, Y.Z., 2018. Investigation on the control effect of Echinococcus in Delingha area. Graziery veterinary sciences,3
57. Wu, W.P., Wang, H., Wang, Q., Zhou, X.N., Wang, L.Y., Zheng, C.J., Cao, J.P., Xiao, N., Wang, N., Zhu, Y.Y., Niu, Y.L., Xue, C.Z., Zeng, X.M., Fang, Q., Han, S., Yu, Q., Yang,S.J., Fu, Q., Bai, X.F., Tian, T., Li, J.J., Zhang, M.Y., Wu, W.T., Zhang, S.S., Hou, Y.Y., Feng, Y., Ma, X., Li, B., Li, F.K., Guo, W.D., Yang, Y.M., Wu, X.L., Jin, X.L., Zhang, H.W., Yu, S.C., 2018. A nationwide sampling survey on echinococcosis in China during 2012-2016.Chinese Journal of Parasitology and Parasitic Diseases,36,1
58. Yu, F.F., Duan, Z.Z., Wang, W., Li, J., 2018. Investigation and analysis of echinococcosis infection of animals in Aksu City.Modern Agricultural Science and Technology,8
59. Zhang, P., Cai, K.H., 2018. Surveillance for dog infection with Echinococcus granulosus in Tianzhu, Gansu, 2011–2016. Disease Surveillance,33,8
60. Zhang, S.M., Zhang, N., Zhao, J.T., 2018.Survey on the prevalence of hydatidosis in Damao Banner.China Health Care & Nutrition,28,36
61. Cadavid Restrepo Angela M., McManus Donald P., Gray Darren J., Barnes Tamsin S., Williams Gail M., Soares Magalhães Ricardo J., Clements Archie C A., 2018. Spatial prediction of the risk of exposure to Echinococcus spp. among schoolchildren and dogs in Ningxia Hui Autonomous Region, People’s Republic of China.Geospatial health,13,1
62. Liu, V.N., Xu, Y.Y., Cadavid-Restrepo Angela M., Lou, Z.Z., Yan, H.B., Li, L., Fu, B.Q., Gray Darren J., Clements Archie A., Barnes Tamsin S., Williams Gail M., Jia, W.Z., McManus Donald P., Yang, Y.R., 2018.Estimating the prevalence of Echinococcus in domestic dogs in highly endemic for Echinococcosis.Infectious diseases of poverty,7,77
63. Liu, X.D., Shang, W.J., Zhao, C.T., Zhang, S.W., Lu, S.L., Wang, Q.H., 2014. Epidemiological survey results of hydatid disease in Gannan Tibetan Autonomous Prefecture, Gansu Province. Chinese Journal of Endemiology 33(6), 619-622.
64. Liu, Y., Guan, W.P., Huang, Y.J, Yang, L., Zou, K.S., 2014. Analysis on the prevalence of echinococcosis in the fourth division of the Xinjiang Production and Construction Corps. Modern Preventive Medicine 41(2), 343-344, 348.
65. Wen, Q.Y., Liu, G.Y., Ma, S.Y., 2014. Analysis of investigation report on monitoring and control of echinococcosis in Beibei City, the tenth division of Xinjiang Corps, 2010~2013. Medical Information, 27(5), 113-114.
66. Yan, C.Y., Chao, S.P., 2013. Analysis of surveillance results of echinococcosis in Bohu County, Xinjiang in 2012. Bulletin of Disease Control & Prevention (China) 28(6), 43+51.
67. Han, F., 2013. Investigation on the infection level and population control knowledge and related behaviors of Echinococcus granulosus in livestock and dogs in Xinjiang Corps.
68. Ma, L.K., Lin, H.L., Nu, S.L.T., Zu, L.H.M.E., Yan, H., Ba, T.L., A, E.D.K., Zhang, Z.Z., Ma, S.C., 2013. Comprehensive prevention and control measures for livestock hydatid disease in Burqin County. Chinese animal husbandry and veterinary abstracts 29(10), 85-86.
69. Ma, Z.G., Zhang, Z.Z., Zhang, X., Yue, C., Shi, B.X., Liu, L.Y., Xue, J., Cui, J.G., Wang, M.G., Se, L.K., 2013. Investigation on the infection of Echinococcus granulosus in livestock and dogs in Chabuchar County, Xinjiang. Bulletin of Disease Control & Prevention(China) 28(6), 16-17+28.
70. Ma, L.W., 2013. Analysis of monitoring results of echinococcosis in Aheqi County, Xinjiang in 2010. Bulletin of Disease Control & Prevention(China) 28(3), 21+23.
71. Ma, Y.L., 2014.Screening and Analysis of Hydatidosis in Hualong County of Qinghai Province in 2012.Journal of Medical Pest Control 30,5
72. Li, P.Y., Han, Y.R., Li, X.N.,Rebiguri Abudouin., Azan Khali., 2014. Epidemiological research and analysis on hydatid disease of Manas county in 2013.Chinese Journal of Health Laboratory Technology 24,19
73. Ma, X.L., 2014. Survey on the prevalence of echinococcosis in Xining City in 2013.Qinghai Medical Journal 44,10
74. Zhao, J.S., Hou, Y.Y., Zhumahong Ruziguri., Zhang, H.T., 2014.Investigation and analysis of Echinococcus granulosus infection in Xinjiang domestic dogs under different production modes.Bulletin of Disease Control & Prevention(China) 29,6
75. Nilamudine, Zulihoumari., Nusra Te., Ma, K.L., Yan, H., Wu, X., Tohoti, Munlik., Experience in epidemic, control and detection of hydatidosis in Hetian area.2014,5
76. Zhao, J.H., Wu, X.L., Ma, R., Fu, Y.R., Feng, Y.L., 2014. Analysis of hydatid disease prevalence survey in Ningxia in 2012.Ningxia Medical Journal 36,4
77. Zheng, B., Wang, D.L., Wang, H., 2014.Investigation and analysis of epidemic situation of hydatidosis in Pingluo County. China Hwalth Care & nutrition 2014,6
78. Zhao, M.K., Yan, L.J., Ren, G.S., 2014. Epidemiological Survey of Hydatidosis in Datong County of Qinghai Province in 2012.Journal of Medical Pest Control 30,4
79. Kestern, F., Qi, X., Tao, J, Feng, X., Mastin A., Craig, P.S., Vuitton, D.A., Duan, X., Chu, X., Zhu, J., Wen, H. Independent evaluation of a canine Echinococcosis Control Programme in Hobukesar County, Xinjiang, China. Acta Tropica 145,1-7.
80. Dao, J., Liu, J.R., LV, M.D., Wang, Z.Y.,2015. Epidemiological Analysis on Echinococcosis in Ganzi Tibetan Autonomous Prefecture of Sichuan Province，1962－2012.Parasitoses and Infectious Diseases,13,73-80."
81. Gazina Analbek, Shahati Tokhdag,2013.Analysis of surveillance results of hydatid disease in Yumin County, Xinjiang from 2010 to 2012.Xinjiang Medical Journal,43,117-118.
82. Nianga only let,2017. Analysis of Epidemiological Investigation Report of Hydatid Disease in Huangnan Prefecture.World Latest Medicine Information,17,210.
83. Kang, Y.X., Zhang,C.J., He, R.F., Gongsang Q.Z., Baima, Y.J., Suolang, W.J., 2018.Investigation of Echinococcus infection in dogs in Tibet Autonomous Region.Chinese Journal of Parasitology and Parasitic Diseases,36,35-37.
84. Zhang,X.Z.,Li,S.F.,2018.Investigation on the control effect of canine fine grain *Echinococcus* disease in Minhe County. Chinese Qinghai Journal of Animal and Veterinary Sciences,48,52+37.
85. Zhu,J.M.,Wang,J.X.,Shen，L.L.,Yuan,X.,Zhang,X.Y.,Xiao,S,S.,Guo,W.,2015.Dog feces hydatid antigen testing result analysis of Tianzhu county during 2008 －2012.Journal of Medical Pest Control,36,26-27.
86. Li,S.S.,2014.Investigation on the infection of Echinococcus granulosus in shepherd dogs in Huanmenmen area.Chinese Qinghai Journal of Animal and Veterinary Sciences,44,25-26.
87. Guo, Z.H., Li, W., Peng, M., Duo, H., Shen, X.Y., Fu, Y., Irie, T.K., Gan, T.T., Kirino, Y.M., Nasu, T.T., Horii, Y.C., Nonaka, N.K., Epidemiological Study and Control Trial of Taeniid Cestode Infection in Farm Dogs in Qinghai Province, China.The Journal of Veterinary Medical Science 76(3),395-400.
88. Zhao, J., Li, S,F., Yang, L.C., 2018. Effect of Praziquantel on Echinococcus granulosus in dogs.Animals Breeding and Feed 06,95-96
89. Zhao, X.Z., Xiang, X.C., Yin, X.H., Chen, H., 2019. Surveillance and analysis of hydatid disease in Longyang District, Baoshan City, Yunnan Province, 2016-2018.Bulletin of Disease Control & Prevention (China) 34(03),73-74.
90. Zhao, F.T., 2019. Investigation and Analysis of Epidemic Situation of Hydatidosis among Animals in Ganzhou District.Journal of Animal Science and Veterinary Medicine 38(02),58-59.
91. Chen, X.Y., Sethival, Y., Usman, I., 2016. Epidemiological survey of Echinococcus granulosus in Kizilsu Kirgiz Autonomous Prefecture, Xinjiang.Chinese Journal of Parasitology and Parasitic Diseases 34(05),409-413.
92. Huang, X.M., Huang, L.Z., Lv, L.F., Jiang, L., Xie, Y.Q., Xu, X.Z., Epidemiological survey of echinococcus in Liyang, Jiangsu, 2013 ～ 2018.ournal of Tropical Diseases and Parasitology 17(03),153-155.
93. Qi, X.W.,Feng, X.H., Kesteren, F.V., Li, H.T., Song, T., Duan, X.Y., Jiang, T., Chu, X.D., Fang, B.B., Zhang, W.B., Lin, R.Y., W, H., Analysis of the current status and related risk factors of echinococcosis infection in Xinjiang and Buxel Mongolian Autonomous County.Chinese Journal of Endemiology 34(1),56-60.
94. Li, B., 2013, Epidemiological investigation and sequence analysis of gene of echinococcus granulosus, 2013.06.01
95. Niu, M., An, Y.G., 2012. Epidemiological investigation of hydatid disease in Sunan Yugu Autonomous County of Gansu Province.Chin J of Clinical Rational Drug Use 5(5B), 45-47
96. Wang, S.Z., Yang, X.B., Lu, Q.X., Yan, Z.Y., 2013. Investigation on the infection of Echinococcus granulosus in Dulan County.Chinese Qinghai Journal of Animal and Veterinary Sciences 43(2), 31-32
97. Li, X.L., Chen, G.L., Huo, Y.M., Yu, Y.L., Li, N., Feng, Y.W., Qiu, C., Tong, H.C., Ji, H.C., Wang, H.T., Zhang, H.Y., Wu, H.Y., Duan, Y.P., Sun, Y.H., Wu, S.M., Dong, J.H., Xia, T., Yuan, J.G., 2018. Epidemiological survey of hydatid disease in the dam area of ​​Chengde City.Heilongjiang Animal Science and Veterinary Medicine 2018(08), 107-109
98. Duan, H.J., Wu, X.L., Qi, R.T., Fu, Y.R., Ma, R., 2019. Analysis of the surveillance results of Echinococcus in Ningxia in 2017.Chin J Parasitol Parasit Dis 37(4), 433-436
99. Li, B.F., Wu, F.W., Yan, X.L., Zi, J.R., Peng, J., Bao, X.Y., Cai, X., Yang, Y.M., 2019. Epidemiological analysis of echinococcus in Yunnan Province from 2012 to 2017.Chin J Parasitol Parasit Dis 37(5), 576-582
100. Li, S.L., 2017. Epidemiology and prevention methods of hydatid disease in Pingchuan District from 2013 to 2017.Chinese Baby 2017(21), 228
101. Fan, L., Pu, F., 2017. Surveillance of echinococcosis in a county from 2010 to 2015.Clinical research 25(8),13-14
102. Ayipu, R., Rehemutula, A., 2017. Investigation on the epidemic situation of echinococcosis in Shawan County, Xinjiang.China Health Care & Nutrition 27(23), 334-335
103. Wang, D.M., He, R.F., Gongsangquzhen., Xiao, D., Suolangwangjie., Xue, L., Bianbazhuoma., Li, J.Z., 2018.Prevalence of echinococcosis in Nyingchi City.Chinese Journal of Parasitology and Parasitic Diseases 36,1.
104. Shi, M.C., Wang, G.J., Zhang, Y.L., Wang, R., Ma, Z.M., Zhao, H.Y., 2015.Evaluation on the control effect of echinococcosis in the fifth division of Xinjiang Construction Corps.Journal of Public Health and Preventive Medicine 26.1
105. Zhan, F., Shi, W.G., Gao, S.Z., Liu, Q.Y., Pan, Y.H., 2019.An epidemiological survey of echinococcosis in livestock in pastoral and semi pastoral areas of Gansu Province.Journal of Traditional Chinese Veterinary Medicine 38.6
106. He, Y.H., Qiang, L.H., 2014.Prevalence of Hydatid Disease in Baiyin City of Gansu in 2011.Chinese Journal of Parasitology and Parasitic Diseases 32.1
107. Hu, Q., He, Z.H., Wang, Y., 2011.Investigation of Echinococcus granulosus infection in dogs in Horqin district, Tongliao City, Inner Mongolia in 2010.Chinese Journal of Endemiology 30.3

Shi, X.Y., Zhao, J., Liu, X.G., Zhang, S.B., Yun, Z.L., 2017.Investigation on the infection and control of dogs and sheep in the high incidence area of hydatidosis in Inner Mongolia.Modern Preventive Medicine 44.12
