# Supplementary material for: Meta-analysis of the prevalence of Echinococcus in dogs in China from 2010 to 2019
Source: PLoS Negl Trop Dis. 2021 Apr 2;15(4):e0009268. doi: 10.1371/journal.pntd.0009268 (PMC8018629; doi:10.1371/journal.pntd.0009268)
Supplement: S4 Table — (DOCX) [file pntd.0009268.s008.docx]

**S4 Table.** Egger’s for publication bias

| slope | bias | se. bias | t | df | P-value |
| --- | --- | --- | --- | --- | --- |
| 0.207 | 2.823 | 2.286 | 1.235 | 106 | 0.220 |
